# P2X_1_, P2X_4_, and P2X_7_ Receptor Knock Out Mice Expose Differential Outcome of Sepsis Induced by α-Haemolysin Producing *Escherichia coli*

**DOI:** 10.3389/fcimb.2017.00113

**Published:** 2017-04-06

**Authors:** Anne-Sofie Greve, Marianne Skals, Steen K. Fagerberg, Wulf Tonnus, Svend Ellermann-Eriksen, Richard J. Evans, Andreas Linkermann, Helle A. Praetorius

**Affiliations:** ^1^Department of Biomedicine, Aarhus UniversityAarhus, Denmark; ^2^Department of Clinical Microbiology, Aarhus University HospitalAarhus, Denmark; ^3^Division of Nephrology, Medical Clinic III, University Hospital Carl Gustav Carus DresdenDresden, Germany; ^4^Department of Molecular and Cell Biology, University of LeicesterLeicester, UK

**Keywords:** sepsis, P2X, caspase-8, uropathogenic, *E. coli*

## Abstract

α-haemolysin (HlyA)-producing *Escherichia coli* commonly inflict severe urinary tract infections, including pyelonephritis, which comprises substantial risk for sepsis. *In vitro*, the cytolytic effect of HlyA is mainly mediated by ATP release through the HlyA pore and subsequent P2X_1_/P2X_7_ receptor activation. This amplification of the lytic process is not unique to HlyA but is observed by many other pore-forming proteins including complement-induced haemolysis. Since free hemoglobin in the blood is known to be associated with a worse outcome in sepsis one could speculate that inhibition of P2X receptors would ameliorate the course of sepsis. Surprisingly, this study demonstrates that ${\text{P}2\text{X}}_{1}^{-/-}$ and ${\text{P}2\text{X}}_{4}^{-/-}$ mice are exceedingly sensitive to sepsis with uropathogenic *E. coli*. These mice have markedly lower survival, higher cytokine levels and activated intravascular coagulation. Quite the reverse is seen in ${\text{P}2\text{X}}_{1}^{-/-}$ mice, which had markedly lower cytokine levels and less coagulation activation compared to controls after exposure to uropathogenic *E. coli*. The high cytokine levels in the ${\text{P}2\text{X}}_{7}^{-/-}$ mouse are unexpected, since P2X_7_ is implicated in caspase-1-dependent IL-1β production. Here, we demonstrate that IL-1β production during sepsis with uropathogenic *E. coli* is mediated by caspase-8, since caspase-8 and RIPK3 double knock out mice show substantially lower cytokine during sepsis and increased survival after injection of TNFα. These data support that P2X_7_ and P2X_4_ receptor activation has a protective effect during severe *E. coli* infection.

## Introduction

Sepsis is the major cause of death in intensive care units worldwide. Urinary tract infections (UTI), often caused by uropathogenic *E. coli*, have been identified as the prime source in ~10–30% of severe sepsis or septic shock (Wagenlehner et al., 2007). Sepsis in general can result in multiple organ failure and death as a consequence of uncontrolled activation of the innate immune system with high circulating levels of pro-inflammatory cytokines such as interleukin 6 (IL-6), IL-8, IL-1β and tumor necrosis factor α (TNFα) (Nupponen et al., 2001; Santana et al., 2015). In addition to cytokines, adenosine triphosphate (ATP) comprises one of many host damage-associated-molecular-patterns (DAMPs) molecules released to the extracellular space during cell injury in response to invasive pathogens (Land et al., 2016; Ousingsawat et al., 2017). The level of extracellular ATP is sensed by P2Y and P2X receptors and serves a wide range of physiological functions including thrombocyte aggregation, taste, pain, and chemo-sensing (Burnstock, 2009). In terms of infection, ATP is a well-recognized signaling molecule released at sites of inflammation/cell injury, and plays a central role in immune cell migration, chemotaxis, and cytokine release (Junger, 2008; Bours et al., 2011). In particular, the P2X_7_ receptor has attracted considerable attention for its importance in immune cell communication (Di Virgilio and Vuerich, 2015), release of pro-inflammatory cytokines (Suzuki et al., 2004; Clark et al., 2010; Shieh et al., 2014; Trubiani et al., 2014), and recruitment of macrophages and lymphocytes (Moncao-Ribeiro et al., 2011; da Silva et al., 2013). Therefore, the P2X_7_ has emerged as a potential anti-inflammatory therapeutic target.

We have previously established that the effect of the pore-forming virulence factor α-haemolysin (HlyA), secreted from certain *E. coli* strains, is mainly secondary to ATP release and P2X receptor activation (Skals et al., 2009). *E. coli* strains that produce HlyA are commonly isolated from patients with severe urinary tract infections (Johnson, 1991; Bien et al., 2012) and contribute to the pathogenesis of urosepsis. *In vitro*, we have demonstrated in erythrocytes that ATP is released immediately after HlyA is inserted into the membrane (Skals et al., 2014) and potentiates haemolysis by activation of mainly P2X_1_ in mice and mainly P2X_7_ receptors in humans (Skals et al., 2009). Importantly, the P2X-dependent amplification of cell damage is not specific to HlyA but is seen in response to many types of cytolytic proteins such as α-toxin from *S. aureus* (Skals et al., 2011), LtxA from *A. actinomycetemcomitans* (Munksgaard et al., 2012), ApxIA haemolysin from *A. pleuropneumoniae* (Masin et al., 2013), β-toxin from *C. perfringens* (Nagahama et al., 2015), and membrane attack complex formed after complement activation (Hejl et al., 2012). Based on this, we speculated that P2X receptor antagonists may ameliorate the symptoms of urosepsis.

Urosepsis is not easily modeled experimentally as installation of bacteria in the urinary tract is unable to produce reproducible septic events in rodents. However, direct injection of uropathogenic bacteria intravenously has been proven to be a beneficial model for urosepsis (Barber et al., 2016). The chosen uropathogenic bacterium (ARD6, O6:K13:H1) commonly causes urinary tract infection in humans and, in addition to HlyA, also expresses other virulence enhancing proteins such as P-fimbriae (Zingler et al., 1992).

In this study, we used a model of acute sepsis in P2X_1_, P2X_4_, and P2X_7_ receptor deficient mice under anesthesia using these uropathogenic *E. coli*, in accordance with Danish legislation for animal research. We establish that mice lacking P2X_7_ and P2X_4_ are significantly more susceptible to sepsis inflicted by uropathogenic *E. coli*. ${\text{P}2\text{X}}_{7}^{-/-}$ and ${\text{P}2\text{X}}_{4}^{-/-}$ mice died more quickly and showed massively increased plasma cytokine levels, intravascular haemolysis and activation of the coagulation system. Strikingly, we found a markedly smaller spleen in ${\text{P}2\text{X}}_{7}^{-/-}$ mice compared to ${\text{P}2\text{X}}_{7}^{+/+}$ even though the spleen just like the control enlarged during sepsis. The ${\text{P}2\text{X}}_{1}^{-/-}$ mice seemed relatively protected against sepsis with uropathogenic *E. coli* with prominently lower plasma cytokine levels. The unexpected, high IL-1β-production in the ${\text{P}2\text{X}}_{7}^{-/-}$ mice is likely to result from P2X_7_-independent activation of caspase-8 (casp8), since casp8/RIPK3 double knock out mice exhibit markedly lower cytokine levels compared to controls during sepsis with uropathogenic *E. coli*. These data support that P2X_7_ and P2X_4_ receptor activation protects against severe infection either by limiting the number of bacteria in the blood or by diminishing the casp8 dependent cytokine storm.

## Materials and methods

### Escherichia coli

The uropathogenic *E. coli* strain ARD6 (serotype: O6:K13:H1) and the non-pathogenic strain D2103 (serotype OR:H48) were obtained from Statens Serum Institute (Copenhagen, Denmark). The bacteria were grown on agar plates containing LB media and kept for up to 1 month at 4°C. For each experiment a fresh liquid preparation of *E. coli* was cultured overnight by transferring one colony to 4 ml LB medium at 37°C at 250 rpm. The following morning, the culture was centrifuged twice and re-suspended in sterile saline. Live and dead bacteria in this preparation were distinguished by a cell viability kit (BD biosciences) and approximately 10% dead cells were present in this type of preparation. *E. coli* was counted by flow cytometry (Accuri C6, BD Biosciences) and different concentrations of bacteria were used depending on the specific mouse strain the protocol used (see section “mouse model of sepsis” below). In all experiments, isolated bacteria were injected into mice via a lateral tail vein in 150 μl saline.

### Animals

*P2X*_7_^−/−^ mice on a balb/cj background (over 10 generation back crossed) were bred at the Institute of Biomedicine, Aarhus University and matched with either ${\text{P}2\text{X}}_{7}^{+/+}$ littermates from heterozygous breeding or balb/cj mice from Janvier Labs (Saint-Berthevin, France). The ${\text{P}2\text{X}}_{7}^{-/-}$ mice were originally developed by GlaxoSmithKline and bred into the balb/cj background. Animal experiments with the P2X antagonists, BBG, were performed on balb/cj mice from Janvier Labs.

*P2X*_1_ *and P2X*_4_ wild type and knockout mice were bred at the Institute of Biomedicine, Aarhus University, by heterozygous breeding and littermates were used. P2X_1_ mice were on a C57BL/6J background and P2X_4_ were on a mixed background (C57BL6.b6129s). All P2X mice used in this study were 8–10 week old males with a weight of 25.1 ± 0.8 g.

*Caspase-8/RIPK-3*^−*DKO*^ mice were bred in the Kiel facility, Germany, as published (Linkermann et al., 2013), and matched with C57BL/6N mice from either Charles River, Sulzfeld or Janvier Labs. The authors would like to thank NR Jorgensen for providing the P2X_7_ mice, J Leipziger for providing the P2X_4_ mice, D Green for providing the casp8/RIPK3^DKO^ (Oberst et al., 2011), V. Dixit and K. Newton (Genentech) for providing RIPK3 deficient mice (Newton et al., 2004) and R Hakim for casp8 heterozygous mice (Salmena et al., 2003).

### Blood samples

Immediately before the mice were euthanised, blood was drawn from the abdominal vena cava into a heparinised syringe and centrifuged at 1,000 g for 10 min to obtain plasma. Plasma was used for measurements of intravascular haemolysis, levels cytokines and thrombin-antithrombin complexes.

### Haemolysis

Haemolysis was measured immediately as the absorbance at 410 nm (dilution 1:32) on a spectrophotometer (Ultraspec III, LKB Biochrom) and the value evaluated by reference curve. The remaining plasma was stored at −20°C for later evaluation of cytokines and levels of thrombin-antithrombin complexes. Plasma was stored and used within 30 days.

### Reagents

Brilliant Blue G (BBG) was from Sigma-Aldrich and NF449 was from Tocris Bioscience (Bristol, UK). Purified murine TNFα was purchased from BioLegend (Uithoorn, Netherlands). All substances were dissolved in sterile isotonic saline (0.9% NaCl). CBA flex sets for measuring cytokines were from BD Biosciences. TAT Complexes Mouse ELISA Kit for measuring levels of thrombin-antithrombin was from Abcam (Cambridge, UK).

### Cytokines

TNF-α, IL-1β, KC (murine equivalent of IL-8 in humans), IL-6 were measured on stored plasma samples (−20°C) on a flow cytometer (BD Accuri C6, BD Biosciences) according to manufactures instructions.

### Thrombin-antithrombin (TAT) complexes

TAT were measured in heparin-anticoagulated plasma samples with TAT Complexes Mouse ELISA Kit according to manufactures instructions.

### Mouse model of sepsis

Sepsis was induced in mice on three different backgrounds (balb/cj, C57BL/6rj and mixed). The number of bacteria required to investigate survival rates within 6 h were adjusted to an optimal number of 165·10^6^ in balb/cj mice. However, we observed that mice on a C57BL/6rj and mixed background required a higher number of bacteria to die within the observation period. Thus, the number of bacteria was increased by a factor 1.5, corresponding to 248·10^6^. These concentrations will be referred to as high doses in the result section. The high doses were decreased by a factor 0.25 corresponding to ~ 41·10^6^ for balb/cj and 62·10^6^ for C57bl/6j and mixed backgrounds and will be referred to as the low doses in the result section. All mice were anesthetized by a subcutaneous injection of ketamine (100 mg kg^−1^) and xylazine (7.5 mg kg^−1^) and placed on a heating plate at 38°C. *E. coli* was injected in a lateral tail vein in a volume of 150 μl sterile saline and mice were monitored carefully for either 2.5 or 6 h according to the protocol used. For both protocols, additional anesthesia was administered approximately every 45 min. Body temperature was measured continuously by a rectal thermometer (Bioseb, Florida, USA). Blood pressure was measured every 30 min by determining the tail blood volume with a volume-pressure recording sensor and an occlusion tail-cuff (Kent Scientific Corporation, Connecticut, USA) and respiratory rate (RR) was visually monitored every 30 min. BBG, NF449 or saline were given 2 h before and 2 and 4 h after *E. coli* injection. BBG was given subcutaneously and NF449 was given *iv*.

The following 3 protocols were used in this study:

*Survival—6 h:* Mice were continuously observed after *E. coli* injection in the tail vain. Mice were given injections of BBG, NF449 or saline. Body temperature, blood pressure and respiratory rate were monitored. 165·10^6^ ARD6 was given to the mice on balb/cj background and 248·10^6^ ARD6 was given to C57BL6 and mixed background. In the following these concentrations will be referred to as the high dose of *E. coli*.

*Harvesting blood and organs—2.5 h—high dose*: Mice were continuously observed after *E. coli* injection and organs and blood were harvested after 2.5 h. Mice were given injections of BBG, NF449 or saline. Body temperature, blood pressure and respiratory rate were monitored. ARD6 (165·10^6^) was given to mice on balb/cj background and 248·10^6^ ARD6 was given to C57BL/6j and mixed background. *Harvesting blood—2.5 h—low dose*: Mice were continuously observed after *E. coli* injection and the blood was harvested after 2.5 h. Body temperature, blood pressure and respiratory rate were monitored. ARD6 (41·10^6^) was given to mice on balb/cj background and 62·10^6^ ARD6 was given to C57BL/6j and mixed background. In the following these concentrations will be referred to as the low dose of *E. coli*.

*TNF*α*-induced shock—*The model of TNFα-induced shock has been described in detail previously (Cauwels et al., 2003). In our experiments, C57BL/6N, RIPK3-deficient and casp8/RIPK3^DKO^ mice received a single *iv-*injection of 25 mg kg^−1^ murine TNFα (in 200 μl PBS) via the tail vein. Animals were under permanent observation and survival was checked every 15 min in accordance to the authorisation of the local committee for the preservation of animals act.

### Colony forming units (CFU)

CFU were determined in blood after the animals were sacrificed. Whole blood (10 μl) was diluted 1/100 and 5 μl was plated on a blood agar plate and cultured overnight at 37°C and the number of colonies were counted and expressed as CFU μl blood^−1^.

### Histology

Organs were isolated after euthanasia. Lungs, liver, spleen, kidneys and heart were immersion fixed in 4% paraformaldehyde for at least 24 h and stored at 4°C until further preparation. For preparation, the organs were dehydrated in a series of three ethanol solutions (70, 96, and 99.9%), xylen and then imbedded in paraffin for haematoxylin eosin (HE) staining.

### Spleen weight

The spleens were dissected free from connecting tissues. The weight of each spleen was determined and the result expressed as percentage of body weight.

### Ethics

The experiments performed in this study were approved by Danish ethic committee for animal research “Dyreforsøgstilsynet” (2014-15-0201-00316) and by the local committee for the preservation of animal act of Christian-Albrechts-University Kiel, Germany.

### Statistics

Statistical analysis was performed using GraphPad Prism software. Survival studies were analyzed by Kaplan-Meier curve and log-rank test. All other data was reported as mean ± SEM and analyzed using Student *t*-test. A *p* < 0.05 was considered statistically significant and marked by ^*^.

## Results

The present study was undertaken to determine the *in vivo* effects of uropathogenic *E. coli* during sepsis. Specifically, we were interested in the role of P2X_1_ and P2X_7_ because these receptors are predominantly responsible for the cytotoxic effects of HlyA *in vitro*. Moreover, we included the P2X_4_ receptor because it is expressed in most bone marrow derived cells and because it is hard to distinguish pharmacologically from P2X_7_. To this end, we used mice deficient of the given receptors and or pharmacological blockage of P2X_1_ and P2X_7_ receptors. Sepsis was induced in anesthetized mice by *iv*-injection of the HlyA-producing and uropathogenic *E. coli* strain ARD6. Mice were kept under anesthesia to follow regulations by the Danish ethic committee for animal research.

### Establishing a sepsis model in mice

Mice exposed to ARD6 develop bacteraemia demonstrated by colony forming units on blood culture. This was not seen in control mice injected with saline. In addition, balb/cj mice subjected to *iv*-injection of a high dose of ARD6 (165·10^6^) showed an increase in body temperature over the observation period of 2.5 h (Figure S1A). Moreover, animals exposed to bacteria developed haematuria and clearly showed acute tubular necrosis. The inner renal medulla showed obvious protein deposits in the lumen of the renal tubules in the animals exposed to ARD6 (Figure S2). Taken together these observations indicate septic shock. To distinguish the effect of uropathogenic *E. coli*, we included a non-pathogenic control strain of *E. coli* (D2103, OR:H48), which in contrast to ARD6 does not cause pyelonephritis in mice after injection into the urinary bladder (unpublished observations). When balb/cj mice were injected with an equal amount D2103 (165·10^6^), all mice survived the 6-h observation period (Figure S3A). Moreover, mice subjected to D2103 did not show intravascular haemolysis and only very slight changes in plasma cytokine levels (Figures S3B,C).

### Role of P2X_7_ receptors in ARD6 sepsis

**Figure 1:**
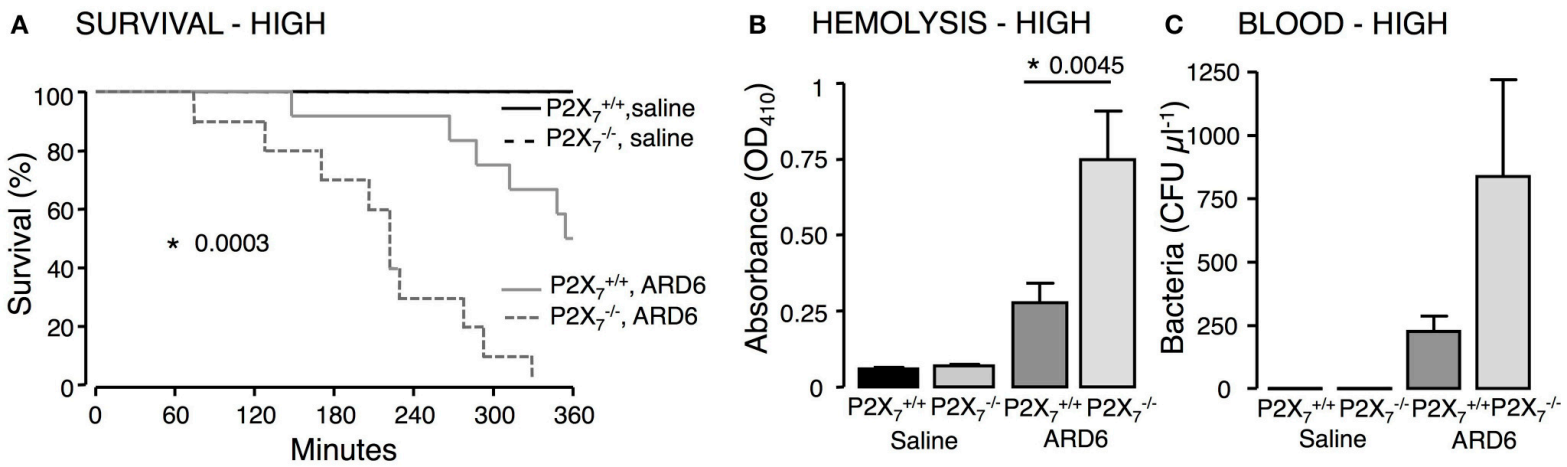
**P2X_7_ deficiency in *E. coli*-induced sepsis**. ARD6 was injected into anesthetized ${\text{P}2\text{X}}_{7}^{+/+}$ and ${\text{P}2\text{X}}_{7}^{-/-}$ mice (high dose—165 million bacteria). **(A)** Kaplan-Meier plot shows survival after ARD6 injection, *n* = 6 for controls and 10–12 for ARD6 for each genotype. **(B)** Absorbance of plasma as an indication of plasma haemolysis 2.5 h after ARD6 injection, *n* = 5 for control and 10–12 for each genotype. **(C)** Colony forming units (CFU) in the blood 2.5 h after ARD6 infection, *n* = 4 for controls and 11 for both genotypes. ^*^p < 0.05.

**Figure 2:**
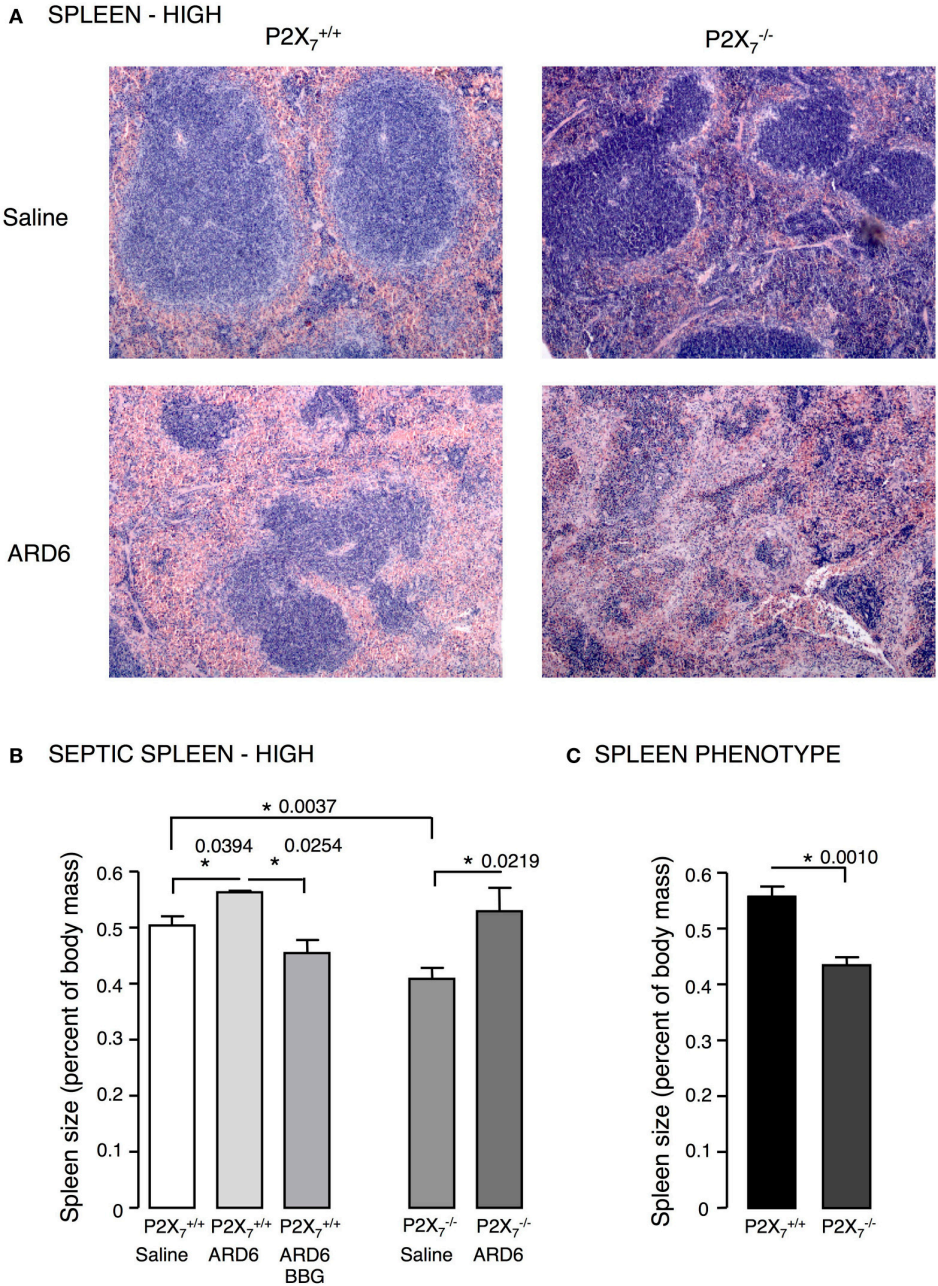
***E. coli*****-induced sepsis—effects on the spleen. (A)** Sections of spleens from P2X_7_^+/+^ or ${\text{P}2\text{X}}_{7}^{-/-}$ mice exposed to either saline or ARD6 (high dose—165 million). ARD6 or saline were injected *iv* and the animals were observed for 2.5 h before they were sacrificed. Organs were harvested and prepared for haematoxylin-eosin (HE) staining. Images are representative of 8 experiments. **(B)** Dissected spleens from ${\text{P}2\text{X}}_{7}^{+/+}$ and ^−/−^ mice after saline, ARD6, or ARD6 and BBG treatment, *n* = 4–8. **(C)** Dissected spleens from untreated ${\text{P}2\text{X}}_{7}^{+/+}$ and ^−/−^ mice, *n* = 8 in both groups. ^*^p < 0.05.

We investigated the role of the P2X_7_ receptor in this sepsis model over a 6-h observation period. Surprisingly, we found a significant reduction in the survival of the ${\text{P}2\text{X}}_{7}^{-/-}$ mice compared to ${\text{P}2\text{X}}_{7}^{+/+}$ (Figure 1A). The average survival time was 323.3 ± 18.5 min for ${\text{P}2\text{X}}_{7}^{+/+}$ and 214.9 ± 24.2 min for ${\text{P}2\text{X}}_{7}^{-/-}$ mice after a high dose of ARD6 (*p* = 0.0027). We also observed a significantly higher intravascular haemolysis in ${\text{P}2\text{X}}_{7}^{-/-}$ mice compared to ${\text{P}2\text{X}}_{7}^{+/+}$ controls (Figure 1B), corresponding to approximately 12 and 3% in ${\text{P}2\text{X}}_{7}^{-/-}$ and ${\text{P}2\text{X}}_{7}^{+/+}$ mice, respectively (Figure S1B). The bacterial load was seemingly, higher in blood drawn from ${\text{P}2\text{X}}_{7}^{-/-}$ mice at the end of the experiment (Figure 1C). This did, however, not reach statistical significance compared to ${\text{P}2\text{X}}_{7}^{+/+}$, because of a large inter-animal variation. This does, however, support that P2X_7_ receptor activation has been associated with increased bacterial macrophage-mediated bacterial clearance during sepsis (Csoka et al., 2015). Animals exposed to bacteria clearly showed acute renal tubular necrosis (Figure S2) in 80% of the mice exposed to ARD6 with no marked difference between ${\text{P}2\text{X}}_{7}^{+/+}$ and ${\text{P}2\text{X}}_{7}^{-/-}$. Haematuria was observed in 90% of the ${\text{P}2\text{X}}_{7}^{-/-}$ mice and 30% of the ${\text{P}2\text{X}}_{7}^{+/+}$ mice following ARD6 exposure. This could be a simple consequence of higher haemolysis in ${\text{P}2\text{X}}_{7}^{-/-}$ mice, however, it potentially suggests damage to the filtration barrier. In support of this notion, amorphous protein was observed in the tubular lumen of 40% ${\text{P}2\text{X}}_{7}^{+/+}$ and 63% ${\text{P}2\text{X}}_{7}^{-/-}$ mice exposed to ARD6; this was most obvious in the renal medulla (Figure S2B). None of the ${\text{P}2\text{X}}_{7}^{+/+}$ or ${\text{P}2\text{X}}_{7}^{-/-}$ mice exposed to saline showed any evidence of tubular necrosis or debris in the tubular lumen (Figure S2). Interestingly, this sepsis model caused quite dramatic changes in the morphology of the spleen (Figure 2A). In the mice injected with saline, the spleen appeared normal, dominated by large blue areas following HE staining of the white pulp. After ARD6 treatment, the ratio between white and red pulp changed markedly, apparently with a reduction of the marginal zone reducing the overall diameter of the white pulp. This could result from mobilization of B and T cells from the spleen during infection, since this has been demonstrated in spleens from septic patients (Hotchkiss et al., 2001; Gunia et al., 2005). However, it could potentially also reflect increased binding and phagocytosis of damaged red blood cells in the red pulp overshadowing the white pulp and thus, changes the ratio between the two. The low white/red pulp is seen in 80% of the spleen from ${\text{P}2\text{X}}_{7}^{+/+}$ mice and in 63% of the spleen from ${\text{P}2\text{X}}_{7}^{-/-}$ mice. This could theoretically suggest that splenic monocytes/macrophages are unable to recognize HlyA-induced erythrocyte damage in ${\text{P}2\text{X}}_{7}^{-/-}$ mice, as previously suggested (Fagerberg et al., 2013). We also measured spleen to body weight ratio and found a substantial increase in spleen mass after injection of ARD6 both in the ${\text{P}2\text{X}}_{7}^{+/+}$ and in ${\text{P}2\text{X}}_{7}^{-/-}$ (Figure 2B). The mean increase was 0.06% of body mass in ${\text{P}2\text{X}}_{7}^{+/+}$ and 0.12% of body mass in ${\text{P}2\text{X}}_{7}^{-/-}$ mice. Mice pre-treated with BBG did not exhibit any enlargement of the spleen upon exposure to ARD6. Remarkably, the saline treated ${\text{P}2\text{X}}_{7}^{-/-}$ mice had a significantly smaller spleen compared to saline-injected ${\text{P}2\text{X}}_{7}^{+/+}$ (Figure 2B). Therefore, we measured spleen sizes in untreated ${\text{P}2\text{X}}_{7}^{+/+}$ and ${\text{P}2\text{X}}_{7}^{-/-}$ mice and found that the ${\text{P}2\text{X}}_{7}^{-/-}$ mouse indeed had a significantly smaller spleen compared to ${\text{P}2\text{X}}_{7}^{+/+}$ (Figure 2C).

**Figure 3:**
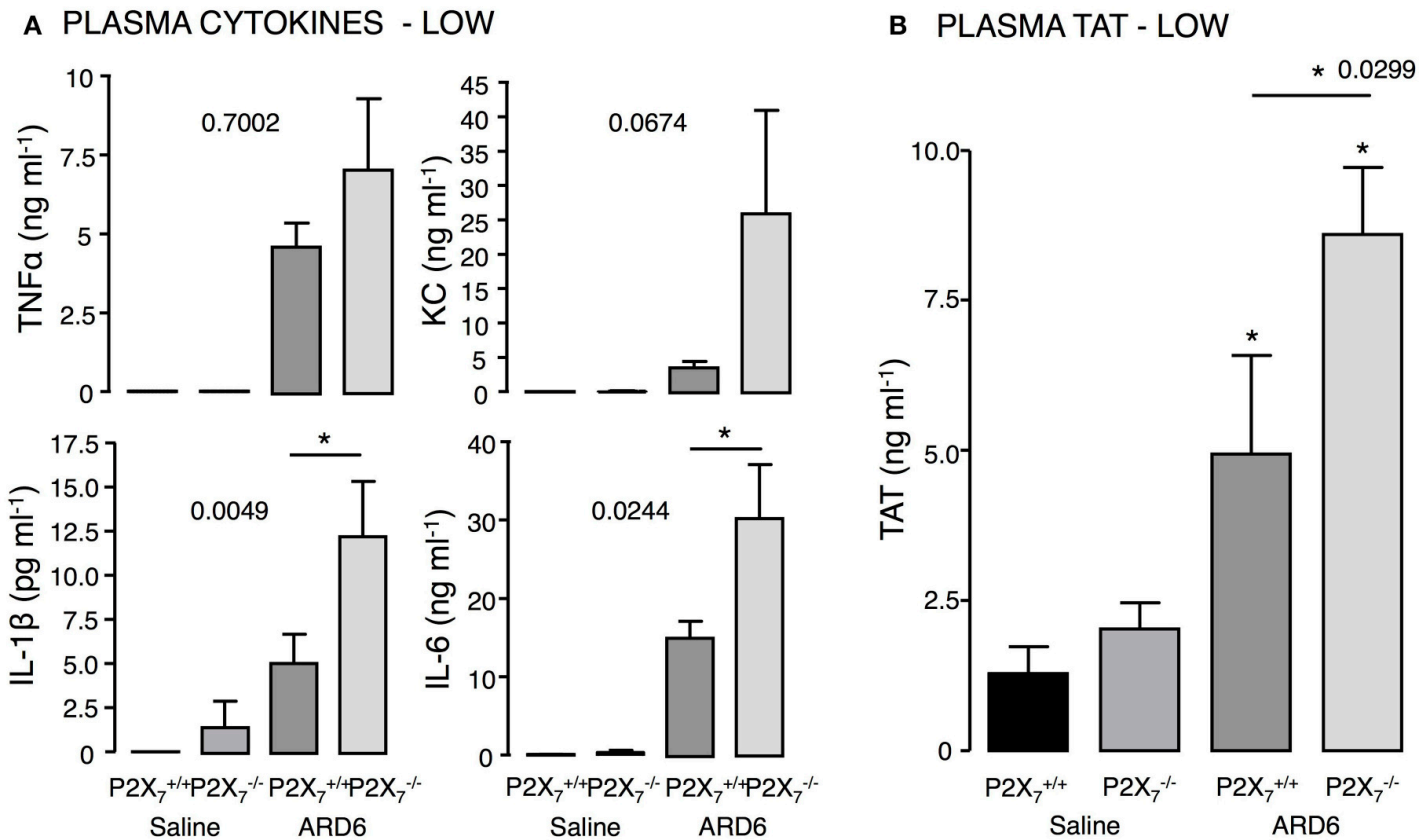
**P2X_7_ deficiency and *E. coli*-induced sepsis**. ARD6 was injected into anesthetized ${\text{P}2\text{X}}_{7}^{+/+}$ and ${\text{P}2\text{X}}_{7}^{-/-}$ mice (low dose—41 million). **(A)** plasma TNFα, KC, IL-1β, and IL-6 measured 2.5 h after ARD6 injection, *n* = 7–8 for controls and 8–9 for each genotype. **(B)** Plasma thrombin-antithrombin (TAT) complexes—measured 2.5 h after ARD6 injection, *n* = 6–7 for controls and 10–11 for both genotypes. ^*^p < 0.05.

Based on the survival curves, one would expect the cytokine levels to differ between the genotypes. However, although the level of TNFα, KC, IL-1β, and IL-6 were highly elevated 2.5 h after administrating 165·10^6^ ARD6, they did not differ between the two genotypes (Figure S4). We speculated that the bacteria load may cause a ceiling effect, and masks any difference in cytokine levels between the genotypes. Therefore, we measured cytokine levels after exposing mice to a reduced dose of ARD6 (41·10^6^). In this situation, IL-6 and IL-1β were distinctly higher in the ${\text{P}2\text{X}}_{7}^{-/-}$ mice compared to controls (Figure 3A), whereas TNFα and KC was not statistically significantly different between the groups. Thus, the higher mortality in the ${\text{P}2\text{X}}_{7}^{-/-}$ mouse is associated with higher cytokine release, supporting previous data that massive cytokine storm is associated with disadvantageous outcome of sepsis (London et al., 2010; Weber et al., 2015).

High cytokine levels are associated with the severity of sepsis and development of disseminated intravascular coagulation (for review see Gando et al., 2016). Interestingly, we found markedly higher levels of thrombin-antithrombin (TAT) complexes in plasma from ${\text{P}2\text{X}}_{7}^{-/-}$ compared to ${\text{P}2\text{X}}_{7}^{+/+}$ mice (Figure 3B), which indicates enhanced activation of the coagulation cascade in these mice. Coincidently, we observed that the buffy coat in blood samples from ${\text{P}2\text{X}}_{7}^{-/-}$ mice exposed to ARD6 was almost un-detectable (data not quantified). This could potentially suggest infection-induced depletion of thrombocytes in ${\text{P}2\text{X}}_{7}^{-/-}$ mice and support the notion of massively activated coagulation system in these mice.

**Figure 4:**
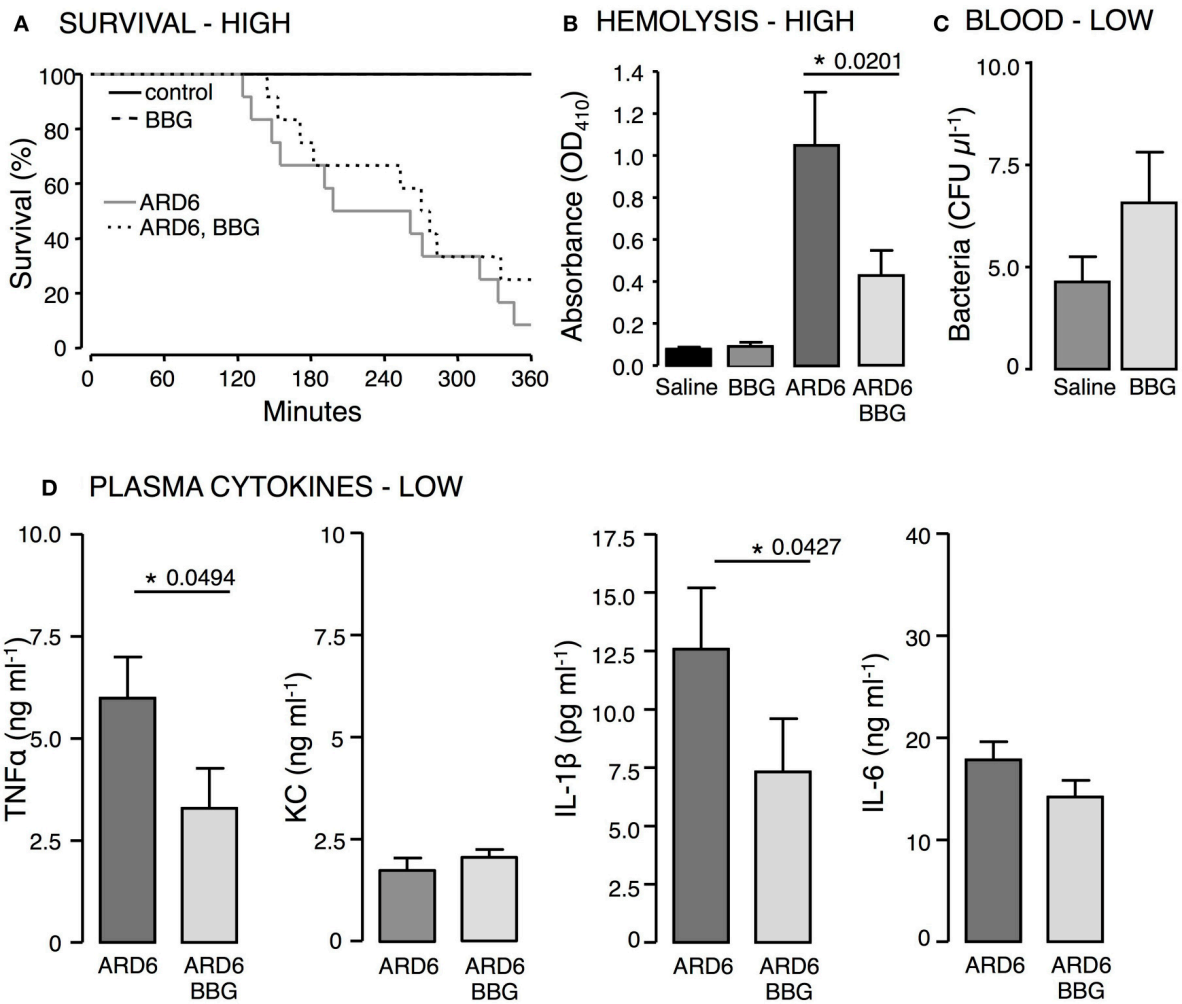
**The effect of P2X_7_ receptor inhibition in *E. coli*-induced sepsis**. ARD6 was injected *iv* into anesthetized balb/cj mice (low dose—41 million). Two hours prior to ARD6 injection the animals were administered the P2X_7_ antagonist BBG or vehicle (50 mg kg^−1^, subcutaneously). **(A)** Kaplan-Meier plot shows survival, *n* = 6 for controls and 10–12 for ARD6 with or without BBG. **(B)** Absorbance of plasma as an indication of plasma haemolysis 2.5 h after ARD6 injection, *n* = 13 for all four groups. **(C)** Colony forming units from blood samples 2.5 h after a low number of bacteria **(D)** Levels of the cytokines TNFα, KC, IL-1β, and IL-6 2.5 h after ARD6 injection, *n* = 14 for ARD6 and 13 for ARD6 + BBG. ^*^p < 0.05.

To support the data from the ${\text{P}2\text{X}}_{7}^{-/-}$ mouse, we tested the well-known P2X antagonist Brilliant Blue G (BBG) that has some selectivity toward P2X_7_ in wild type balbc/j mice. BBG was chosen because the color allows us to directly measure the antagonist concentration in plasma. BBG was given subcutaneously (50 mg kg^−1^) 2 h prior to the *iv*-injection of ARD6 and the mice were observed for 6 h under anesthesia, subsequently surviving mice were culled. At the end of the 6 h period the plasma level of BBG was determined to be 1.2 μM, well above the 1 μM needed to block P2X_7_ receptors. BBG treated mice showed a tendency toward an increased survival rate but this was not statistically significant (Figure 4A). Similar to the *in vitro* experiments (Skals et al., 2009), BBG inhibited haemolysis *in vivo* (Figure 4B). BBG had no statistically significant effect on the number of bacteria in the blood (Figure 4C) but caused a significant decrease in TNFα and IL-1β after injection of 41 million ARD6 and no significant effect on KC or IL-6 (Figure 4D). Thus, pre-treatment with BBG does not mimic the phenotype of the P2X_7_ receptor deficient mice.

### Role of P2X_1_ and P2X_4_ in ARD6 sepsis

BBG is not completely selective for P2X_7_ receptors, and has been shown to antagonize both P2X_1_ and P2X_4_ receptors (Jiang et al., 2000; Seyffert et al., 2004). This may potentially explain the discrepancy between the ${\text{P}2\text{X}}_{7}^{-/-}$ mice and the wild type mice treated with BBG. Therefore, we compared the outcome of sepsis in P2X_1_ and P2X_4_ deficient mice.

**Figure 5:**
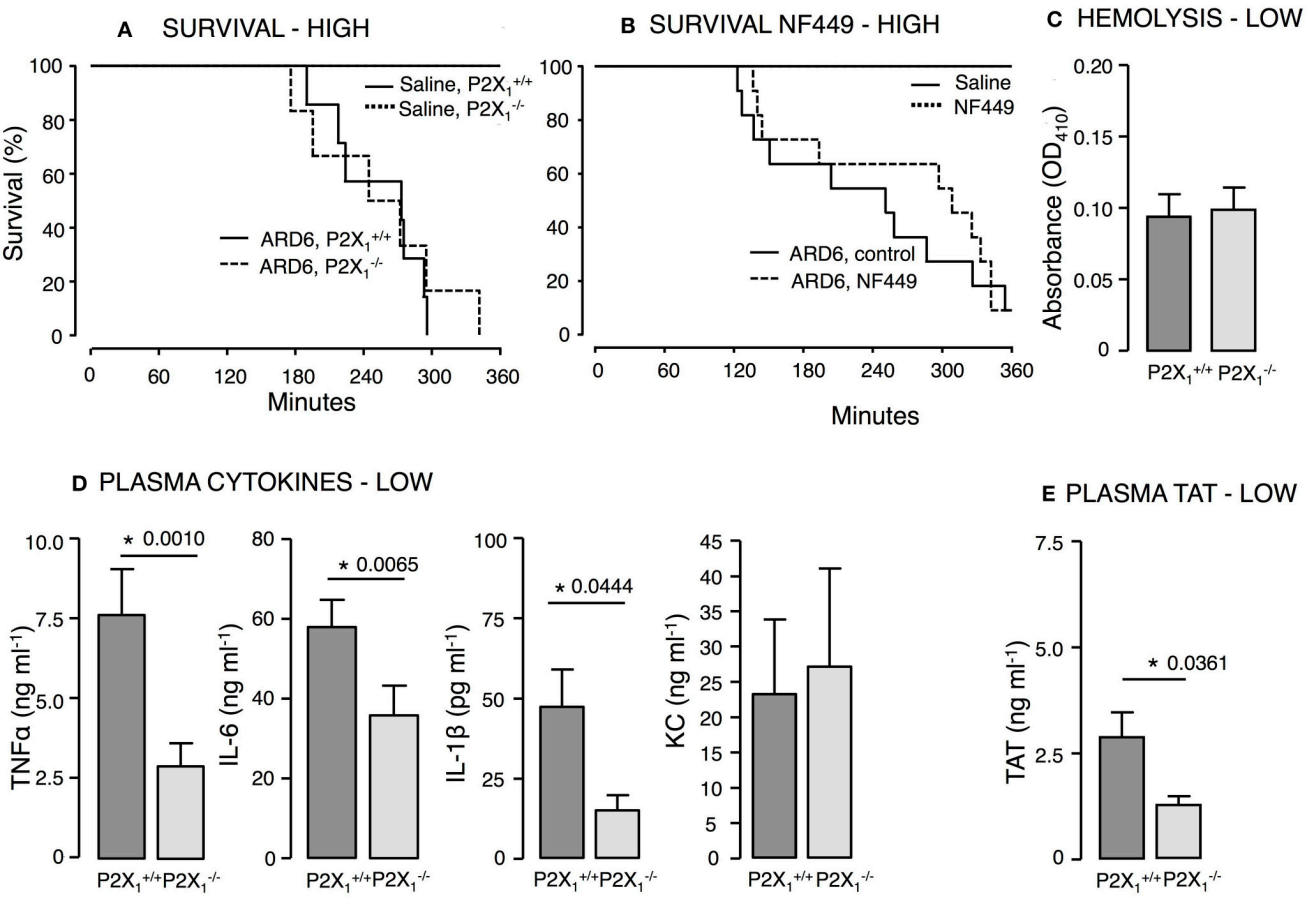
**P2X_1_ deficiency and P2X_1_ inhibition in *E. coli*-induced sepsis**. ARD6 was injected into anesthetized ${\text{P}2\text{X}}_{1}^{+/+}$ and ${\text{P}2\text{X}}_{1}^{-/-}$ mice at a concentration of either low (61 million) or high (248 million) number of bacteria. **(A)** Kaplan-Meier plot shows survival (high dose), *n* = 6 for control and *n* = 12 for both genotypes. **(B)** Survival in the presence or absence of the P2X_1_ antagonist NF449 (100 mg kg^−1^, *iv*) in balb/c mice. The mice were exposed to the high concentration of bacteria, *n* = 6 for control with or without NF449 and *n* = 10–12 for ARD6 with or without NF449. **(C)** Absorbance of plasma as an indication of plasma haemolysis after the low dose, *n* = 8 for both genotypes **(D)** Levels of thrombin-antithrombin (TAT) complexes in plasma 2.5 h after the low dose, *n* = 8 for both genotypes. **(E)** Levels of TNFα, KC, IL-1β, and IL-6 2.5 h after the low dose of ARD6, *n* = 13 both genotypes. ^*^p < 0.05.

Mice on a C57BL/6 background required a markedly higher number of bacteria to develop lethal sepsis within the observation period. We did not observe any difference in mortality between ${\text{P}2\text{X}}_{1}^{+/+}$ and ${\text{P}2\text{X}}_{1}^{-/-}$ mice after a high dose of ARD6 (Figure 5A). On average, however, the ${\text{P}2\text{X}}_{1}^{-/-}$ survived 57 min longer compared to the ${\text{P}2\text{X}}_{1}^{+/+}$. The P2X_1_ antagonist NF449 showed a tendency toward an increase in survival, but this was not statistically significant (Figure 5B). It must be noted that NF449 is degraded quickly (Hechler et al., 2005) and thus, may not provide full P2X_1_R inhibition during the experiment. In a parallel series of experiments in ${\text{P}2\text{X}}_{1}^{+/+}$ and ${\text{P}2\text{X}}_{1}^{-/-}$ mice, intravascular haemolysis, TAT and cytokine levels were measured in plasma 2.5 h after a low dose of ARD6. Intriguingly, TNFα, IL-1β, and IL-6 and TAT levels were all lower in the ${\text{P}2\text{X}}_{1}^{-/-}$ compared to ${\text{P}2\text{X}}_{1}^{+/+}$ (Figures 5D,E), whereas intravascular haemolysis was similar in the two genotypes (Figure 5C). Notably, P2X_1_ receptor expression has a positive impact on the cytokine storm inflicted by the bacterial infection and thus, any P2X_1_antagonizing effect of BBG may potentially be responsible for the lower cytokine levels observed during sepsis in animals pre-exposed to BBG.

**Figure 6:**
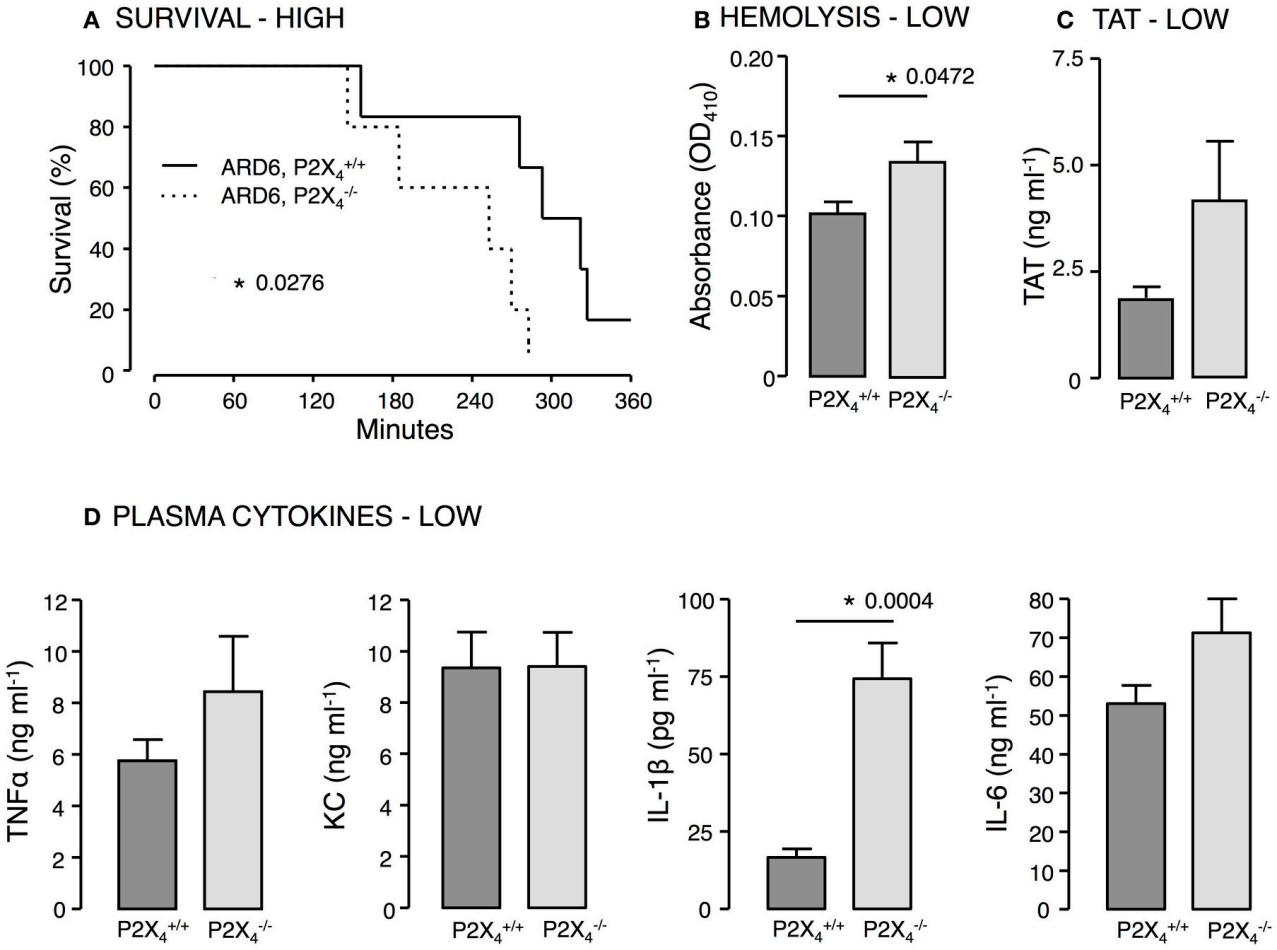
**P2X_4_ deficiency in *E. coli*-induced sepsis**. ARD6 was injected into anesthetized ${\text{P}2\text{X}}_{4}^{+/+}$ and ${\text{P}2\text{X}}_{4}^{-/-}$ mice at a concentration of either low (61 million) or high (248 million) bacteria. **(A)** Kaplan-Meier plot shows survival after the high dose, *n* = 5 for each genotypes **(B)** Level of haemolysis after the low dose, *n* = 8 for ${\text{P}2\text{X}}_{4}^{+/+}$ and 6 for ${\text{P}2\text{X}}_{4}^{-/-}$. **(C)** Levels of thrombin-antithrombin (TAT) complexes in plasma 2.5 h after the low dose of bacteria, *n* = 8 for each genotype. **(D)** Levels of TNFα, KC, IL-1β, and IL-6 2.5 h after the low dose of ARD6, *n* = 11 both genotypes. ^*^p < 0.05.

*P2X*_4_—Similar to our findings in ${\text{P}2\text{X}}_{7}^{-/-}$ mice, ${\text{P}2\text{X}}_{4}^{-/-}$ mice showed decreased survival when exposed to a high dose of ARD6 compared to ${\text{P}2\text{X}}_{4}^{+/+}$ controls (Figure 6A). Moreover, we found higher intravascular haemolysis, plasma IL-1β levels as well as higher TAT levels in the ${\text{P}2\text{X}}_{4}^{-/-}$ after a low ARD6 dose compared to control (Figures 6B–D). Thus, the data on this knock out mouse in many ways resembles data obtained in the ${\text{P}2\text{X}}_{7}^{-/-}$ mice and supports the notion that mice with high cytokine levels in plasma are more prone to die of sepsis. These data also support that mice deficient in P2X_4_ and P2X_7_ receptor are more sensitive to acute severe infection and that this sensitivity is not a result of reduced immune reaction.

### Casp8 and receptor-interacting-protein-3 (RIPK3) in ARD6 sepsis and TNFα shock

**Figure 7:**
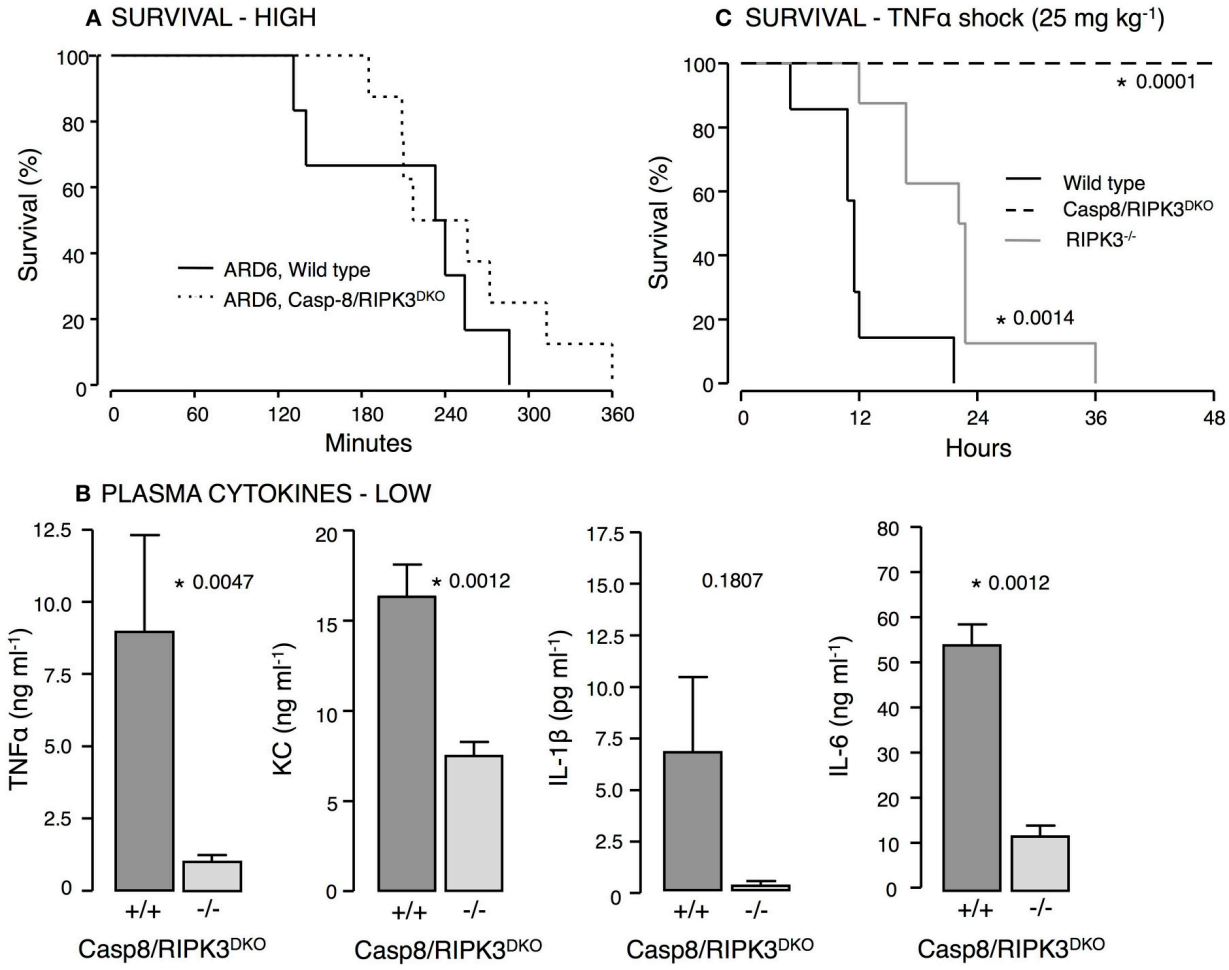
**Casp8 and RIPK3 deficiency in *E. coli*-induced sepsis and TNFα-induced shock**. ARD6 was injected into anesthetized wild type and casp8/RIPK3^DKO^ mice at a low (61 million) or high concentration (248 million). **(A)** Kaplan-Meier plot shows survival after the high dose, *n* = 6 for wild type and 8 for casp8/RIPK3^DKO^. **(B)** Levels of TNFα, KC, IL-1β and IL-6 2.5 h after the low dose of ARD6, *n* = 7 for wild type and 6 for casp8/RIPK3^DKO^. **(C)** Kaplan-Meier plot shows survival after TNFα shock (25 mg kg^−1^) was induced in wildtype, RIPK3 deficient and casp8/RIPK3^DKO^, *n* = 8 in all three groups. ^*^p < 0.05.

IL-1β production is surprisingly prominent in both ${\text{P}2\text{X}}_{7}^{-/-}$ and ${\text{P}2\text{X}}_{4}^{-/-}$ mice exposed to uropathogenic *E. coli*. This underscores that IL-1β production in this model can occur P2X_7_R-independently. Numerous studies have indicated that the NOD-like receptor family pyrin domain containing 3 (NLRP3) inflammasome can be activated by casp8 leading to pro-IL-1β processing (Gringhuis et al., 2012; Gurung et al., 2014; Antonopoulos et al., 2015). Interestingly, activation of casp8 can occur P2X_7_ receptor independently (Felley et al., 2016) and thus, may explain the IL-1β production in the ${\text{P}2\text{X}}_{7}^{-/-}$ mice. Casp8 deficiency in mice is embryonically lethal (Varfolomeev et al., 1998) because casp8 suppresses receptor-interacting protein kinase 3 (RIPK3) (Kang et al., 2013), which cause massive inflammasome activation in Casp8^−/−^ mice. Mice lacking both casp8 and RIPK3 are, however, viable (Kaiser et al., 2011; Oberst et al., 2011) and showed similar survival during 6-h observation after *iv*-injection of ARD6 (high dose, Figure 7A). Strikingly, the cytokine levels were substantially lower in casp8/RIPK3^DKO^ mice compared to controls following a low dose of ARD6 (Figure 7B). Therefore, we tested how these mice manage a less severe stimulus and mimicked severe inflammatory response syndrome (SIRS) by intravenous injection of TNFα (25 μg kg^−1^). In these experiments the mice were not anesthetised during the procedure and were carried out at Christian-Albrechts-University Kiel, Germany under German legislation for animal experiments and in accordance with the local committee for the preservation of animal act. This procedure resulted in death of all wild type mice within 36 h, whereas casp8/RIPK3^DKO^ mice showed a striking 100% survival (Figure 7C). RIPK3 deficiency alone did not protect mice from dying of TNFα injection although they showed a marginal increase in survival compared to wild type. These data suggest an important upstream involvement of casp8 in this model. We conclude that P2X-receptors are crucial determinants for the outcome of sepsis induced by HlyA-producing *E. coli* in mice. The increased immunoreactivity in P2X_7_ and P2X_4_ deficient mice is likely to be mediated *via* non-canonical activation of IL-1β via casp8.

## Discussion

Urinary infections are exceedingly common and often caused by *E. coli*, known as the dominant facultative bacterial agent in the normal intestinal flora. However, simple urinary infections can progress to severe pyelonephritis and sepsis. The invasive, more aggressive *E. coli*-strains responsible for these severe infections are serotypically distinct from facultative strains and frequently produce the virulence factor α-haemolysin (Cavalieri et al., 1984; Bhakdi et al., 1988). A recent study showed that specific fine-tuning of HlyA expression by the human cystitis isolate UTI189 alters the course of both acute and chronic urinary tract infection in mice (Nagamatsu et al., 2015). In the present study, we investigated acute sepsis in mice induced by an uropathogenic α-haemolysin producing *E. coli* strain (ARD6). We chose to induce sepsis by direct injection of live bacteria intravenously in mice, since this model allow us to specifically choose the sepsis-causing bacterium. The virulence factor HlyA is known to cause severe cell damage in a P2X-receptor dependent fashion and thus, we were interested in the role of three P2X receptors (P2X_1_, P2X_4_, and P2X_7_) in sepsis caused by HlyA-producing, uropathogenic *E. coli*.

IL-1β is a key cytokine in sepsis during which the plasma levels of the cytokine can become exceedingly high (for review see Dinarello, 2005). The NLRP3 inflammasome is essential in processing and activation of IL-1β during inflammation (Lamkanfi and Dixit, 2014) and a decrease in intracellular K^+^ concentration is a prime activator of the inflammasome (Cain et al., 2001; Munoz-Planillo et al., 2013). Stimulation of ionotropic P2X_7_Rs on macrophages by ATP directly causes the K^+^ efflux and maturation and release of IL-1β (Perregaux and Gabel, 1994; Kahlenberg and Dubyak, 2004; Lister et al., 2007; Qu et al., 2007; Pelegrin et al., 2008; Wiley et al., 2011). These findings prompted the concept that P2X_7_R antagonists potentially could be used against inflammatory diseases of the kidney, in rheumatoid arthritis and pain disorders (Arulkumaran et al., 2011; Alves et al., 2013). Here, surprisingly we demonstrate that ${\text{P}2\text{X}}_{7}^{-/-}$ mice have an enhanced susceptibility to sepsis induced by uropathogenic *E. coli* with an increased mortality compared to wild type mice. The coagulation cascade is often activated alongside the immune system during sepsis and results in thrombin formation and platelet activation (for review see Brass, 2003). Thrombocytopenic mice are associated with a higher systemic bacterial load, increased plasma levels of pro-inflammatory cytokines (TNFα, IL-6, and IFN-γ) and accordingly a poorer outcome of sepsis induced by Gram-negative bacteria (van den Boogaard et al., 2015). In the present study, we found an increased plasma level of TAT-complexes in ${\text{P}2\text{X}}_{7}^{-/-}$ mice, which is associated with increase mortality during sepsis (van den Boogaard et al., 2015). Increased mortality and increased TAT were also found in the P2X_4_ deficient mice. Thus, one may speculate that thrombocytopenia could be a part of an immune deficiency in ${\text{P}2\text{X}}_{7}^{-/-}$ and ${\text{P}2\text{X}}_{4}^{-/-}$ mice. Moreover, high plasma concentration of hemoglobin during sepsis is also associated with increased mortality (Larsen et al., 2010; Adamzik et al., 2012) and ${\text{P}2\text{X}}_{7}^{-/-}$ mice showed increased plasma levels of hemoglobin compared to wild type. Thus, several risk factors associated with poorer outcome was observed in our murine model of urosepsis.

Previous studies from our group demonstrate a sizable reduction of HlyA-induced cell damage by P2X receptor antagonists on human and murine erythrocytes (Skals et al., 2009, 2014) and monocytes (Fagerberg et al., 2016). Therefore, it was exceedingly surprising to find that ${\text{P}2\text{X}}_{7}^{-/-}$ mice were not relatively protected, but rather distinctively more sensitive ARD6-induced sepsis. This contrasts with *in vivo* studies that have shown improved survival of ${\text{P}2\text{X}}_{7}^{-/-}$ mice when sepsis was induced by coecal ligation and puncture (Santana et al., 2015), lipopolysaccharide (LPS)-injection (Yang et al., 2015) and adenovirus infection (Lee et al., 2012). However, like in the present study, decreased survival and a greater bacterial load in the blood of ${\text{P}2\text{X}}_{7}^{-/-}$ mice subjected to coecal ligation and puncture has been reported (Csoka et al., 2015). Thus, P2X_7_ receptors have a critical role during sepsis and potential survival of this critical condition is a fine balance of the degree of inflammasome-activation and subsequent cytokine production. Massive infection apparently reveals an immune deficiency in ${\text{P}2\text{X}}_{7}^{-/-}$ mice, a notion generally supported by the markedly smaller spleen in ${\text{P}2\text{X}}_{7}^{-/-}$ mice. Our study clearly reveals acute splenomegaly in septic mice, which is explained through a combination of the severe infection and resultant intravascular erythrocyte damage. The tentatively higher infection-induced splenomegaly in the ${\text{P}2\text{X}}_{7}^{-/-}$ fits the lower survival rates, higher cytokine levels and intravascular haemolysis. Interestingly, a recent study showed clear up-regulation of the NLRP3 inflammasome pathway in primary microglial and macrophages from ${\text{P}2\text{X}}_{7}^{-/-}$ mice (Franceschini et al., 2015). Such an up-regulation may explain the higher cytokine levels and the increased mortality in ${\text{P}2\text{X}}_{7}^{-/-}$ mice if the NLRP3 inflammasome could be activated by an alternative pathway to casp-1. It must be noted that neither of the available ${\text{P}2\text{X}}_{7}^{-/-}$ mice are complete knockouts (Nicke et al., 2009; Masin et al., 2012). This is the result of the many splice variations of the P2X_7_ receptor. The splice variant still remaining in the ${\text{P}2\text{X}}_{7}^{-/-}$ mice used in the current study is expressed in the spleen (Nicke et al., 2009). It is, however, unlikely that the difference between ${\text{P}2\text{X}}_{7}^{-/-}$ and the P2X_7_ antagonism would result from this splice variation, because the mice in which some P2X_7_ receptor function is preserved show a worse outcome compared to general inhibition of the receptor.

Strikingly, the P2X receptor antagonist BBG, did not reduce the survival of sepsis induced by uropathogenic *E. coli*. On the contrary, BBG showed a tendency toward prolonging survival of infected mice and statistically significantly reduce plasma IL-1β and TNFα following a low dose of ARD6. BBG was given at a dose, which resulted in a plasma concentration known to give maximal inhibition of both HlyA and complement induced haemolysis in mice (Hejl et al., 2012). Essentially, one could imagine that acute inhibition of P2X_7_ may have other consequences. However, although BBG is primarily used as a P2X_7_antagonist, it also inhibits P2X_1_ and P2X_4_ receptors (Jiang et al., 2000; Seyffert et al., 2004). Thus, if either P2X_1_ or P2X_4_ receptors oppose the effect of P2X_7_ in sepsis, it may potentially explain the discrepancy between the ${\text{P}2\text{X}}_{7}^{-/-}$ and the results obtained with BBG. Interestingly, we found that specific lack of P2X_1_ receptors considerably reduced level of the cytokines TNFα, IL-1β, and IL-6 and lowered coagulation activation following the low dose of ARD6. Notably, the P2X_1_ mouse is on a C57BL/6 background, which has a loss of function mutation in the P2X_7_, at least in the splice variant expressed in T lymphocytes (Adriouch et al., 2002) and is deficient in NLRP-1 (Boyden and Dietrich, 2006), which both may oppose the effect of P2X_1_ deficiency. Two recent studies where sepsis-like conditions were induced in ${\text{P}2\text{X}}_{1}^{-/-}$ mice show contradicting results. One study showed increased survival in ${\text{P}2\text{X}}_{1}^{-/-}$ mice after LPS injection (10 mg kg^−1^) (Maitre et al., 2015), while another demonstrated decreased survival after injection of 20 mg kg^−1^ LPS (Lecut et al., 2012) in mice lacking P2X_1_ receptors. Since both these studies also had ${\text{P}2\text{X}}_{1}^{-/-}$ on a C57BL/6 background and thus, a less functioning P2X_7_ receptor, this may explain the higher sensitivity in the mice at higher dose of LPS. In mice pre-treated with NF449 there was no difference in intravascular haemolysis or cytokine levels in plasma (data not shown), which is likely to result from inadequate blockage of the receptor as previously suggested from the substance pharmacokinetics (Hechler et al., 2005). Thus, there may be room for a specific P2X_1_ antagonist with a prolonged effect in the supportive therapy of septic conditions, since it is very likely that the effect of BBG is mediated through P2X_1_ receptor inhibition.

The P2X_4_ receptor is also known to be expressed in monocytes and macrophages (Kawano et al., 2006) and to influence cytokine release and cell death via P2X_7_-dependent mechanisms (Kawano et al., 2006; Perez-Flores et al., 2015). Thus, activation of the P2X_4_ receptor in macrophages may have similar effects as the P2X_7_ receptor in terms of macrophage function including IL-1β release and apoptosis. This study supports a similar function of P2X_7_ and P2X_4_ receptors in the acute septic response. Similar to the P2X_7_ receptor, ${\text{P}2\text{X}}_{4}^{-/-}$ mice died earlier upon exposure to HlyA-producing *E. coli* and showed an increased level of IL-1β. Though the finding from ${\text{P}2\text{X}}_{4}^{-/-}$ mice cannot explain the discrepancy between the ${\text{P}2\text{X}}_{7}^{-/-}$ and BBG data, these data point to an important function of P2X_4_Rs in acute severe infection.

The question is; what causes the high cytokine levels, particularly of IL-1β in ${\text{P}2\text{X}}_{7}^{-/-}$ mice? As previously mentioned P2X_7_ has a central role in activation of casp1 and the final cleavage of pro-IL-1β to the active form. However, natural killer cells can induce P2X_7_ receptor-independent monocyte IL-1β release via activation of both casp1 and casp8 (Felley et al., 2016). Casp8 plays an essential role during apoptosis, necroptosis and NLRP3 activation (Mocarski et al., 2011) and has recently been demonstrated to cause cytokine release in a murine model of LPS-induced shock (Oliva-Martin et al., 2016). Mice deficient of casp8 are not viable, which is most likely because casp8 normally suppresses RIPK3 depended necroptosis. Thus, we used the viable casp8/RIPK3^DKO^ and demonstrate that the levels of TNFα, KC, IL-6, and IL-1β were markedly suppressed compared to wild type controls. Despite that we only observed a tendency toward increased survival after this acute, massive infection with uropathogenic *E. coli*, a marked effect of lack of casp8 was observed, if we used a milder model of septic shock—injection of TNFα. Thus, casp8 is readily activated during sepsis induced by HlyA-producing *E. coli* and this pathway may very well explain the cytokine production in the P2X_7_ deficient mice.

In summary, our data demonstrate enhanced susceptibility to sepsis with HlyA-producing *E. coli* in mice lacking P2X_7_ and P2X_4_ receptors, whereas mice lacking P2X_1_ receptors exhibit lower cytokine levels during this condition. Elevated plasma levels of the pro-inflammatory cytokines, free hemoglobin and activation of the coagulation system could potentially explain the poorer outcome of sepsis in the ${\text{P}2\text{X}}_{7}^{-/-}$ and ${\text{P}2\text{X}}_{4}^{-/-}$ mice. Interestingly, this sepsis model strongly activates the non-canonical inflammasome pathway via casp8 short-circuiting the classical P2X_7_ dependent activation of casp1. Deficiency of this pathway completely prevents the infection induced cytokine response. These surprising and new results provide an additional insight into the pathogenesis in sepsis and for new ways to approach the condition pharmacologically.

## Author contributions

Conceived and designed the experiments: AG, MS, AL, and HP. Performed the experiments: AG, MS, SF, WT, AL. Analyzed the data: AG, AL, and MS. Wrote the paper: mainly MS and HP, with contribution from AG, RE, SE, and AL.

### Conflict of interest statement

The authors declare that the research was conducted in the absence of any commercial or financial relationships that could be construed as a potential conflict of interest.

## References

[B1] Adamzik M. Hamburger T. Petrat F. Peters J. de Groot H. Hartmann M. (2012). Free hemoglobin concentration in severe sepsis: methods of measurement and prediction of outcome. Crit. Care 16, R125. 10.1186/cc11425 22800762 PMC3580706

[B2] Adriouch S. Dox C. Welge V. Seman M. Koch-Nolte F. Haag F. (2002). Cutting edge: a natural P451L mutation in the cytoplasmic domain impairs the function of the mouse P2X7 receptor. J. Immunol. 169, 4108–4112. 10.4049/jimmunol.169.8.4108 12370338

[B3] Alves L. A. Bezerra R. J. Faria R. X. Ferreira L. G. da Silva Frutuoso V. (2013). Physiological roles and potential therapeutic applications of the P2X7 receptor in inflammation and pain. Molecules 18, 10953–10972. 10.3390/molecules180910953 24013409 PMC6270334

[B4] Antonopoulos C. Russo H. M. El Sanadi C. Martin B. N. Li X. Kaiser W. J.. (2015). Caspase-8 as an effector and regulator of NLRP3 inflammasome signaling. J. Biol. Chem. 290, 20167–20184. 10.1074/jbc.M115.652321 26100631 PMC4536427

[B5] Arulkumaran N. Unwin R. J. Tam F. W. (2011). A potential therapeutic role for P2X7 receptor (P2X7R) antagonists in the treatment of inflammatory diseases. Expert Opin. Investig. Drugs 20, 897–915. 10.1517/13543784.2011.578068 21510825 PMC3114873

[B6] Barber A. E. Norton J. P. Wiles T. J. Mulvey M. A. (2016). Strengths and limitations of model systems for the study of urinary tract infections and related pathologies. Microbiol. Mol. Biol. Rev. 80, 351–367. 10.1128/MMBR.00067-15 26935136 PMC4867371

[B7] Bhakdi S. Mackman N. Menestrina G. Gray L. Hugo F. Seeger W.. (1988). The hemolysin of *Escherichia coli*. Eur. J. Epidemiol. 4, 135–143. 10.1007/BF00144740 3042445

[B8] Bien J. Sokolova O. Bozko P. (2012). Role of uropathogenic *Escherichia coli* virulence factors in development of urinary tract infection and kidney damage. Int. J. Nephrol. 2012:681473. 10.1155/2012/681473 22506110 PMC3312279

[B9] Bours M. J. Dagnelie P. C. Giuliani A. L. Wesselius A. Di Virgilio F. (2011). P2 receptors and extracellular ATP: a novel homeostatic pathway in inflammation. Front. Biosci. (Schol. Ed). 3, 1443–1456. 10.2741/s235 21622280

[B10] Boyden E. D. Dietrich W. F. (2006). Nalp1b controls mouse macrophage susceptibility to anthrax lethal toxin. Nat. Genet. 38, 240–244. 10.1038/ng1724 16429160

[B11] Brass L. F. (2003). Thrombin and platelet activation. Chest 124(3 Suppl.), 18S–25S. 10.1378/chest.124.3_suppl.18s 12970120

[B12] Burnstock G. (2009). Purinergic signalling: past, present and future. Braz. J. Med. Biol. Res. 42, 3–8. 10.1590/S0100-879X2008005000037 18853040

[B13] Cain K. Langlais C. Sun X. M. Brown D. G. Cohen G. M. (2001). Physiological concentrations of K+ inhibit cytochrome c-dependent formation of the apoptosome. J. Biol. Chem. 276, 41985–41990. 10.1074/jbc.M107419200 11553634

[B14] Cauwels A. Janssen B. Waeytens A. Cuvelier C. Brouckaert P. (2003). Caspase inhibition causes hyperacute tumor necrosis factor-induced shock via oxidative stress and phospholipase A2. Nat. Immunol. 4, 387–393. 10.1038/ni914 12652297

[B15] Cavalieri S. J. Bohach G. A. Snyder I. S. (1984). *Escherichia coli* α-hemolysin: characteristics and probable role in pathogenicity. Microbiol. Rev. 48, 326–343. 6394977 10.1128/mr.48.4.326-343.1984 PMC373222

[B16] Clark A. K. Staniland A. A. Marchand F. Kaan T. K. McMahon S. B. Malcangio M. (2010). P2X7-dependent release of interleukin-1β and nociception in the spinal cord following lipopolysaccharide. J. Neurosci. 30, 573–582. 10.1523/JNEUROSCI.3295-09.2010 20071520 PMC2880485

[B17] Csoka B. Nemeth Z. H. Toro G. Idzko M. Zech A. Koscso B.. (2015). Extracellular ATP protects against sepsis through macrophage P2X7 purinergic receptors by enhancing intracellular bacterial killing. FASEB J. 29, 3626–3637. 10.1096/fj.15-272450 26060214 PMC4550379

[B18] da Silva G. L. Sperotto N. D. Borges T. J. Bonorino C. Takyia C. M. Coutinho-Silva R.. (2013). P2X7 receptor is required for neutrophil accumulation in a mouse model of irritant contact dermatitis. Exp. Dermatol. 22, 184–188. 10.1111/exd.12094 23489421

[B19] Dinarello C. A. (2005). Interleukin-1β. Crit. Care Med. 33, S460–S462. 10.1097/01.CCM.0000185500.11080.91 16340421

[B20] Di Virgilio F. Vuerich M. (2015). Purinergic signaling in the immune system. Auton. Neurosci. 191, 117–123. 10.1016/j.autneu.2015.04.011 25979766

[B21] Fagerberg S. K. Jakobsen M. R. Skals M. Praetorius H. A. (2016). Inhibition of P2X receptors protects human monocytes against damage by leukotoxin from *Aggregatibacter actinomycetemcomitans* and α-hemolysin from *Escherichia coli*. Infect. Immun. 84, 3114–3130. 10.1128/IAI.00674-16 27528275 PMC5067745

[B22] Fagerberg S. Skals M. Leipziger J. Praetorius H. (2013). P2X receptor-dependent erythrocyte damage by α-hemolysin from *Escherichia coli* triggers phagocytosis by THP-1 cells. Toxins 5, 472–487. 10.3390/toxins5030472 23462688 PMC3705273

[B23] Felley L. E. Sharma A. Theisen E. Romero-Masters J. C. Sauer J. D. Gumperz J. E. (2016). Human invariant NKT cells induce IL-1β secretion by peripheral blood monocytes via a P2X7-independent pathway. J. Immunol. 197, 2455–2464. 10.4049/jimmunol.1600790 27534556 PMC5011004

[B24] Franceschini A. Capece M. Chiozzi P. Falzoni S. Sanz J. M. Sarti A. C.. (2015). The P2X7 receptor directly interacts with the NLRP3 inflammasome scaffold protein. FASEB J. 29, 2450–2461. 10.1096/fj.14-268714 25690658

[B25] Gando S. Levi M. Toh C. H. (2016). Disseminated intravascular coagulation. Nat. Rev. Dis. Primers 2, 16037 10.1038/nrdp.2016.37 27250996

[B26] Gringhuis S. I. Kaptein T. M. Wevers B. A. Theelen B. van der Vlist M. Boekhout T.. (2012). Dectin-1 is an extracellular pathogen sensor for the induction and processing of IL-1β via a noncanonical caspase-8 inflammasome. Nat. Immunol. 13, 246–254. 10.1038/ni.2222 22267217

[B27] Gunia S. Albrecht K. May M. Stosiek P. (2005). The white pulp in the setting of the septic spleen caused by different bacteria: a comparative morphometric study. APMIS 113, 675–682. 10.1111/j.1600-0463.2005.apm_262.x 16309426

[B28] Gurung P. Anand P. K. Malireddi R. K. Vande Walle L. Van Opdenbosch N. Dillon C. P.. (2014). FADD and caspase-8 mediate priming and activation of the canonical and noncanonical Nlrp3 inflammasomes. J. Immunol. 192, 1835–1846. 10.4049/jimmunol.1302839 24453255 PMC3933570

[B29] Hechler B. Magnenat S. Zighetti M. L. Kassack M. U. Ullmann H. Cazenave J. P. (2005). Inhibition of platelet functions and thrombosis through selective or nonselective inhibition of the platelet P_2_ receptors with increasing doses of NF_449_ [4,4',4”,4”'-(carbonylbis(imino-5,1,3-benzenetriylbis-(carbonylimino)))tetrakis -benzene-1,3-disulfonic acid octasodium salt]. J. Pharmacol. Exp. Ther. 314, 232–243. 10.1124/jpet.105.084673 15792995

[B30] Hejl J. L. Skals M. Leipziger J. Praetorius H. A. (2012). P2X receptor stimulation amplifies complement-induced haemolysis. Pflugers Arch. 465, 529–541. 10.1007/s00424-012-1174-z 23149487

[B31] Hotchkiss R. S. Tinsley K. W. Swanson P. E. Schmieg R. E. Jr. Hui J. J. Chang K. C.. (2001). Sepsis-induced apoptosis causes progressive profound depletion of B and CD4^+^ T lymphocytes in humans. J. Immunol. 166, 6952–6963. 10.4049/jimmunol.166.11.6952 11359857

[B32] Jiang L. H. Mackenzie A. B. North R. A. Surprenant A. (2000). Brilliant blue G selectively blocks ATP-gated rat P2X7 receptors. Mol. Pharmacol. 58, 82–88. 10.1124/mol.58.1.82 10860929

[B33] Johnson J. R. (1991). Virulence factors in *Escherichia coli* urinary tract infection. Clin. Microbiol. Rev. 4, 80–128. 10.1128/CMR.4.1.80 1672263 PMC358180

[B34] Junger W. G. (2008). Purinergic regulation of neutrophil chemotaxis. Cell. Mol. Life Sci. 65, 2528–2540. 10.1007/s00018-008-8095-1 18463789 PMC4214070

[B35] Kahlenberg J. M. Dubyak G. R. (2004). Mechanisms of caspase-1 activation by P2X7 receptor-mediated K^+^ release. Am. J. Physiol. Cell Physiol. 286, C1100–C1108. 10.1152/ajpcell.00494.2003 15075209

[B36] Kaiser W. J. Upton J. W. Long A. B. Livingston-Rosanoff D. Daley-Bauer L. P. Hakem R.. (2011). RIP3 mediates the embryonic lethality of caspase-8-deficient mice. Nature 471, 368–372. 10.1038/nature09857 21368762 PMC3060292

[B37] Kang T. B. Yang S. H. Toth B. Kovalenko A. Wallach D. (2013). Caspase-8 blocks kinase RIPK3-mediated activation of the NLRP3 inflammasome. Immunity 38, 27–40. 10.1016/j.immuni.2012.09.015 23260196

[B38] Kawano S. Otsu K. Kuruma A. Shoji S. Yanagida E. Muto Y.. (2006). ATP autocrine/paracrine signaling induces calcium oscillations and NFAT activation in human mesenchymal stem cells. Cell Calcium 39, 313–324. 10.1016/j.ceca.2005.11.008 16445977

[B39] Lamkanfi M. Dixit V. M. (2014). Mechanisms and functions of inflammasomes. Cell 157, 1013–1022. 10.1016/j.cell.2014.04.007 24855941

[B40] Land W. G. Agostinis P. Gasser S. Garg A. D. Linkermann A. (2016). DAMP - induced allograft and tumor rejection: the circle is closing. Am. J. Transplant. 16, 3338–3361. 10.1111/ajt.14012 27529775

[B41] Larsen R. Gozzelino R. Jeney V. Tokaji L. Bozza F. A. Japiassu A. M.. (2010). A central role for free heme in the pathogenesis of severe sepsis. Sci. Transl. Med. 2, 51ra71. 10.1126/scitranslmed.3001118 20881280

[B42] Lecut C. Faccinetto C. Delierneux C. van Oerle R. Spronk H. M. Evans R. J.. (2012). ATP-gated P2X1 ion channels protect against endotoxemia by dampening neutrophil activation. J. Thromb. Haemost. 10, 453–465. 10.1111/j.1538-7836.2011.04606.x 22212928

[B43] Lee B. H. Hwang D. M. Palaniyar N. Grinstein S. Philpott D. J. Hu J. (2012). Activation of P2X7 receptor by ATP plays an important role in regulating inflammatory responses during acute viral infection. PLoS ONE 7:e35812 10.1371/journal.pone.0035812 22558229 PMC3338466

[B44] Linkermann A. Brasen J. H. Darding M. Jin M. K. Sanz A. B. Heller J. O.. (2013). Two independent pathways of regulated necrosis mediate ischemia-reperfusion injury. Proc. Natl. Acad. Sci. U.S.A. 110, 12024–12029. 10.1073/pnas.1305538110 23818611 PMC3718149

[B45] Lister M. F. Sharkey J. Sawatzky D. A. Hodgkiss J. P. Davidson D. J. Rossi A. G.. (2007). The role of the purinergic P2X7 receptor in inflammation. J. Inflamm. (Lond). 4:5. 10.1186/1476-9255-4-5 17367517 PMC1838907

[B46] London N. R. Zhu W. Bozza F. A. Smith M. C. Greif D. M. Sorensen L. K.. (2010). Targeting Robo4-dependent slit signaling to survive the cytokine storm in sepsis and influenza. Sci. Transl. Med. 2, 23ra19. 10.1126/scitranslmed.3000678 20375003 PMC2875996

[B47] Maitre B. Magnenat S. Heim V. Ravanat C. Evans R. J. de la Salle H.. (2015). The P2X1 receptor is required for neutrophil extravasation during lipopolysaccharide-induced lethal endotoxemia in mice. J. Immunol. 194, 739–749. 10.4049/jimmunol.1401786 25480563

[B48] Masin J. Fiser R. Linhartova I. Osicka R. Bumba L. Hewlett E. L.. (2013). Differences in purinergic amplification of osmotic cell lysis by the pore-forming RTX toxins *Bordetella pertussis* CyaA and *Actinobacillus pleuropneumoniae* ApxIA: the role of pore size. Infect. Immun. 81, 4571–4582. 10.1128/IAI.00711-13 24082076 PMC3837988

[B49] Masin M. Young C. Lim K. Barnes S. J. Xu X. J. Marschall V.. (2012). Expression, assembly and function of novel C-terminal truncated variants of the mouse P2X7 receptor: Re-evaluation of P2X7 knockouts. Br. J. Pharmacol. 165, 978–993. 10.1111/j.1476-5381.2011.01624.x 21838754 PMC3312493

[B50] Mocarski E. S. Upton J. W. Kaiser W. J. (2011). Viral infection and the evolution of caspase 8-regulated apoptotic and necrotic death pathways. Nat. Rev. Immunol. 12, 79–88. 10.1038/nri3131 22193709 PMC4515451

[B51] Moncao-Ribeiro L. C. Cagido V. R. Lima-Murad G. Santana P. T. Riva D. R. Borojevic R.. (2011). Lipopolysaccharide-induced lung injury: role of P2X7 receptor. Respir. Physiol. Neurobiol. 179, 314–325. 10.1016/j.resp.2011.09.015 21982752

[B52] Munksgaard P. Vorup-Jensen T. Reinholdt J. Soderstrom C. Poulsen K. Leipziger J. (2012). Leukotoxin from *Aggregatibacter actinomycetemcomitans* causes shrinkage and P2X receptor-dependent lysis of human erythrocytes. Cell. Microbiol. 4, 1904–1920. 10.1111/cmi.12021 22906303

[B53] Munoz-Planillo R. Kuffa P. Martinez-Colon G. Smith B. L. Rajendiran T. M. Nunez G. (2013). K^+^ efflux is the common trigger of NLRP3 inflammasome activation by bacterial toxins and particulate matter. Immunity 38, 1142–1153. 10.1016/j.immuni.2013.05.016 23809161 PMC3730833

[B54] Nagahama M. Seike S. Shirai H. Takagishi T. Kobayashi K. Takehara M.. (2015). Role of P2X7 receptor in *Clostridium perfringens* beta-toxin-mediated cellular injury. Biochim. Biophys. Acta 1850, 2159–2167. 10.1016/j.bbagen.2015.08.011 26299247

[B55] Nagamatsu K. Hannan T. J. Guest R. L. Kostakioti M. Hadjifrangiskou M. Binkley J.. (2015). Dysregulation of *Escherichia coli* alpha-hemolysin expression alters the course of acute and persistent urinary tract infection. Proc. Natl. Acad. Sci. U.S.A. 112, E871–E880. 10.1073/pnas.1500374112 25675528 PMC4345586

[B56] Newton K. Sun X. Dixit V. M. (2004). Kinase RIP3 is dispensable for normal NF-kappa Bs, signaling by the B-cell and T-cell receptors, tumor necrosis factor receptor 1, and toll-like receptors 2 and 4. Mol. Cell. Biol. 24, 1464–1469. 10.1128/MCB.24.4.1464-1469.2004 14749364 PMC344190

[B57] Nicke A. Kuan Y. H. Masin M. Rettinger J. Marquez-Klaka B. Bender O.. (2009). A functional P2X7 splice variant with an alternative transmembrane domain 1 escapes gene inactivation in P2X7 knock-out mice. J. Biol. Chem. 284, 25813–25822. 10.1074/jbc.M109.033134 19546214 PMC2757983

[B58] Nupponen I. Andersson S. Jarvenpaa A. L. Kautiainen H. Repo H. (2001). Neutrophil CD11b expression and circulating interleukin-8 as diagnostic markers for early-onset neonatal sepsis. Pediatrics 108:E12. 10.1542/peds.108.1.e12 11433091

[B59] Oberst A. Dillon C. P. Weinlich R. McCormick L. L. Fitzgerald P. Pop C.. (2011). Catalytic activity of the caspase-8-FLIP(L) complex inhibits RIPK3-dependent necrosis. Nature 471, 363–367. 10.1038/nature09852 21368763 PMC3077893

[B60] Oliva-Martin M. J. Sanchez-Abarca L. I. Rodhe J. Carrillo-Jimenez A. Vlachos P. Herrera A. J.. (2016). Caspase-8 inhibition represses initial human monocyte activation in septic shock model. Oncotarget 7, 37456–37470. 10.18632/oncotarget.9648 27250033 PMC5122324

[B61] Ousingsawat J. Cabrita I. Wanitchakool P. Sirianant L. Krautwald S. Linkermann A.. (2017). Ca2+ signals, cell membrane disintegration, and activation of TMEM16F during necroptosis. Cell. Mol. Life Sci. 74, 173–181. 10.1007/s00018-016-2338-3 27535660 PMC11107605

[B62] Pelegrin P. Barroso-Gutierrez C. Surprenant A. (2008). P2X7 receptor differentially couples to distinct release pathways for IL-1beta in mouse macrophage. J. Immunol. 180, 7147–7157. 10.4049/jimmunol.180.11.7147 18490713

[B63] Perez-Flores G. Levesque S. A. Pacheco J. Vaca L. Lacroix S. Perez-Cornejo P.. (2015). The P2X7/P2X4 interaction shapes the purinergic response in murine macrophages. Biochem. Biophys. Res. Commun. 467, 484–490. 10.1016/j.bbrc.2015.10.025 26456657

[B64] Perregaux D. Gabel C. A. (1994). Interleukin-1 beta maturation and release in response to ATP and nigericin. Evidence that potassium depletion mediated by these agents is a necessary and common feature of their activity. J. Biol. Chem. 269, 15195–15203. 8195155

[B65] Qu Y. Franchi L. Nunez G. Dubyak G. R. (2007). Nonclassical IL-1β secretion stimulated by P2X7 receptors is dependent on inflammasome activation and correlated with exosome release in murine macrophages. J. Immunol. 179, 1913–1925. 10.4049/jimmunol.179.3.1913 17641058

[B66] Salmena L. Lemmers B. Hakem A. Matysiak-Zablocki E. Murakami K. Au P. Y.. (2003). Essential role for caspase 8 in T-cell homeostasis and T-cell-mediated immunity. Genes Dev. 17, 883–895. 10.1101/gad.1063703 12654726 PMC196031

[B67] Santana P. T. Benjamim C. F. Martinez C. G. Kurtenbach E. Takiya C. M. Coutinho-Silva R. (2015). The P2X7 receptor contributes to the development of the exacerbated inflammatory response associated with sepsis. J. Innate Immun. 7, 417–427. 10.1159/000371388 25675986 PMC6738781

[B68] Seyffert C. Schmalzing G. Markwardt F. (2004). Dissecting individual current components of co-expressed human P2X1 and P2X7 receptors. Curr. Top. Med. Chem. 4, 1719–1730. 10.2174/1568026043387160 15579104

[B69] Shieh C. H. Heinrich A. Serchov T. van Calker D. Biber K. (2014). P2X7-dependent, but differentially regulated release of IL-6, CCL2, and TNF-α in cultured mouse microglia. Glia 62, 592–607. 10.1002/glia.22628 24470356

[B70] Skals M. Bjaelde R. G. Reinholdt J. Poulsen K. Vad B. S. Otzen D. E.. (2014). Bacterial RTX toxins allow acute ATP release from human erythrocytes directly through the toxin pore. J. Biol. Chem. 289, 19098–19109. 10.1074/jbc.M114.571414 24860098 PMC4081947

[B71] Skals M. G. Jorgensen N. R. Leipziger J. Praetorius H. A. (2009). α-hemolysin from *Escherichia coli* uses endogenous amplification through P2X receptor activation to induce hemolysis. Proc. Natl. Acad. Sci. U.S.A. 106, 4030–4035. 10.1073/pnas.0807044106 19225107 PMC2656199

[B72] Skals M. Leipziger J. Praetorius H. A. (2011). Haemolysis induced by α-toxin from *Staphylococcus aureus* requires P2X receptor activation. Pflugers Arch. 462, 669–679. 10.1007/s00424-011-1010-x 21847558

[B73] Suzuki T. Hide I. Ido K. Kohsaka S. Inoue K. Nakata Y. (2004). Production and release of neuroprotective tumor necrosis factor by P2X7 receptor-activated microglia. J. Neurosci. 24, 1–7. 10.1523/JNEUROSCI.3792-03.2004 14715932 PMC6729576

[B74] Trubiani O. Horenstein A. L. Caciagli F. Caputi S. Malavasi F. Ballerini P. (2014). Expression of P2X7 ATP receptor mediating the IL8 and CCL20 release in human periodontal ligament stem cells. J. Cell. Biochem. 115, 1138–1146. 10.1002/jcb.24756 24851271

[B75] van den Boogaard F. E. Schouten M. de Stoppelaar S. F. Roelofs J. J. Brands X. Schultz M. J.. (2015). Thrombocytopenia impairs host defense during murine *Streptococcus pneumoniae* pneumonia. Crit. Care Med. 43, e75–e83. 10.1097/ccm.0000000000000853 25627210

[B76] Varfolomeev E. E. Schuchmann M. Luria V. Chiannilkulchai N. Beckmann J. S. Mett I. L.. (1998). Targeted disruption of the mouse Caspase 8 gene ablates cell death induction by the TNF receptors, Fas/Apo1, and DR3 and is lethal prenatally. Immunity 9, 267–276. 10.1016/S1074-7613(00)80609-3 9729047

[B77] Wagenlehner F. M. Weidner W. Naber K. G. (2007). Optimal management of urosepsis from the urological perspective. Int. J. Antimicrob. Agents 30, 390–397. 10.1016/j.ijantimicag.2007.06.027 17728107

[B78] Weber G. F. Chousterman B. G. He S. Fenn A. M. Nairz M. Anzai A.. (2015). Interleukin-3 amplifies acute inflammation and is a potential therapeutic target in sepsis. Science 347, 1260–1265. 10.1126/science.aaa4268 25766237 PMC4376966

[B79] Wiley J. S. Sluyter R. Gu B. J. Stokes L. Fuller S. J. (2011). The human P2X7 receptor and its role in innate immunity. Tissue Antigens 78, 321–332. 10.1111/j.1399-0039.2011.01780.x 21988719

[B80] Yang D. He Y. Munoz-Planillo R. Liu Q. Nunez G. (2015). Caspase-11 requires the pannexin-1 channel and the purinergic P2X7 pore to mediate pyroptosis and endotoxic shock. Immunity 43, 923–932. 10.1016/j.immuni.2015.10.009 26572062 PMC4795157

[B81] Zingler G. Ott M. Blum G. Falkenhagen U. Naumann G. Sokolowska-Kohler W.. (1992). Clonal analysis of *Escherichia coli* serotype O6 strains from urinary tract infections. Microb. Pathog. 12, 299–310. 10.1016/0882-4010(92)90048-S 1352840

