# Supplementary material for: P2X1, P2X4, and P2X7 Receptor Knock Out Mice Expose Differential Outcome of Sepsis Induced by α-Haemolysin Producing Escherichia coli
Source: Front Cell Infect Microbiol. 2017 Apr 6;7:113. doi: 10.3389/fcimb.2017.00113 (PMC5382212; doi:10.3389/fcimb.2017.00113)
Supplement: Supplementary file 1 [file Presentation1.PDF]

Supplemental Figures

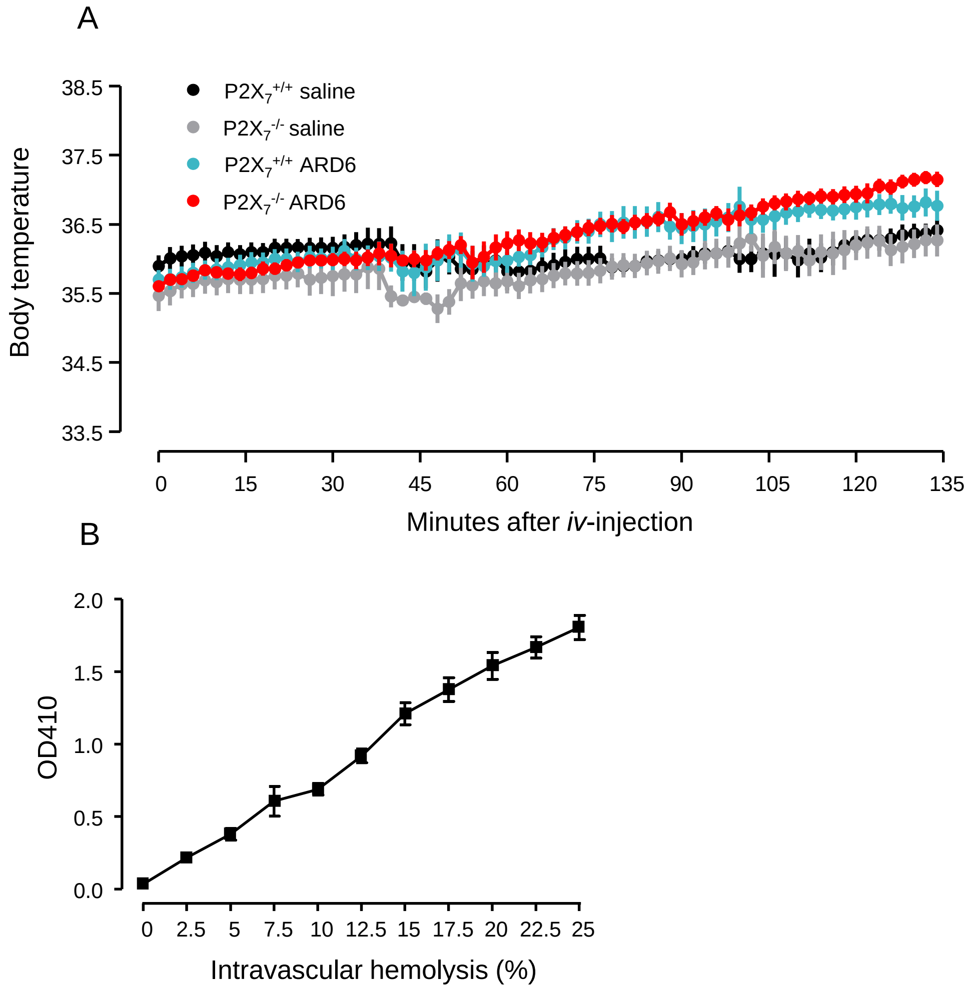

Figure S1. (A) Body temperature measured in mice following injection of ARD6 (high dose) or saline in $P2X_7^{+/+}$ and $P2X_7^{-/-}$ mice. n=7-8 for all 4 groups. (B) Relationship between absorbance (OD410) and intravascular hemolysis. n=7.

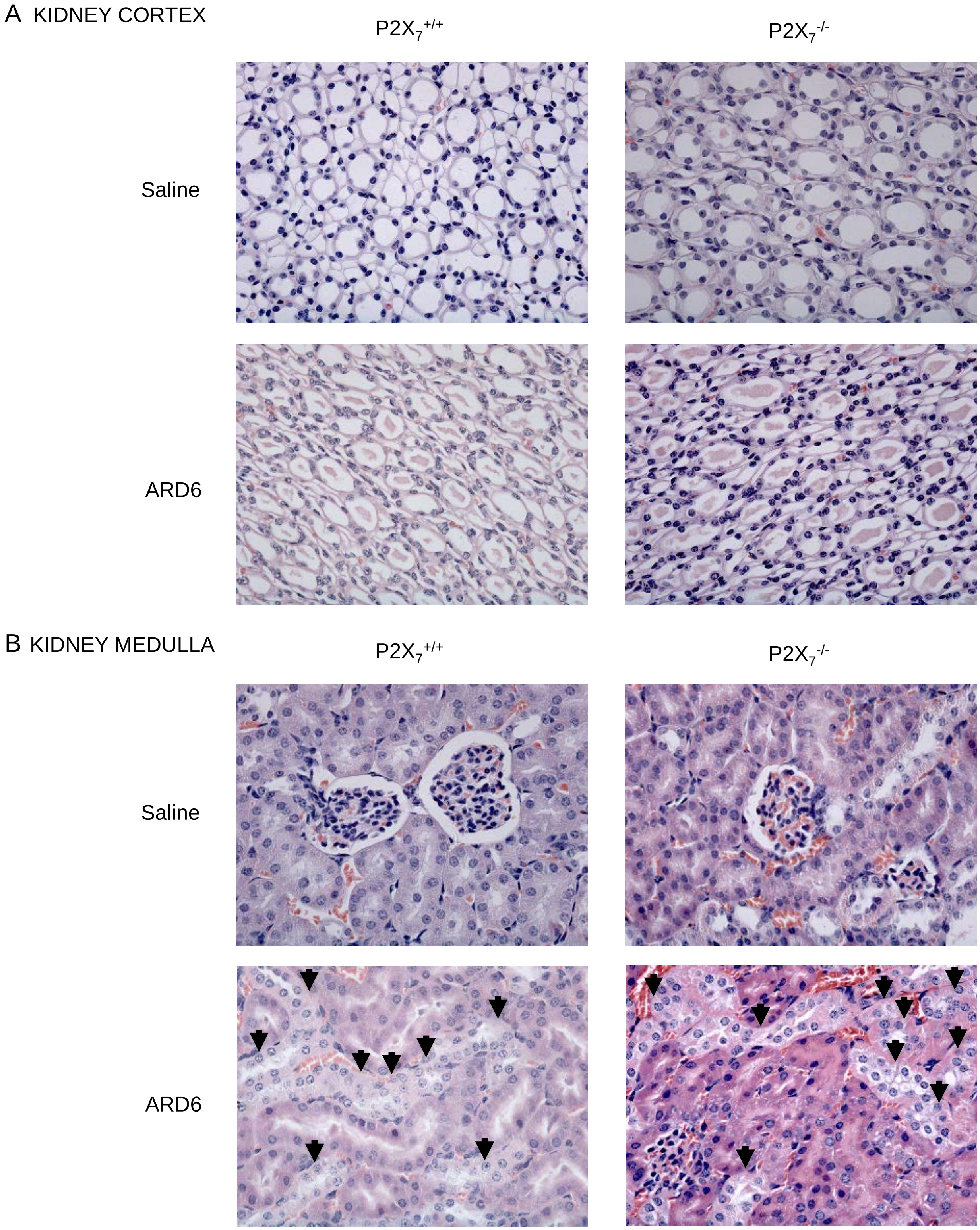

Figure S2. *E. coli*-induced sepsis – effects in the kidney.
Sections of (A) kidney cortex and (B) kidney medulla from $P2X_7^{+/+}$ or $P2X_7^{-/-}$ mice exposed to either saline or high number of ARD6 (165 million). ARD6 or saline were injected *iv* and the animals were observed for 2.5 hours before they were sacrificed. Arrows indicate affected proximal tubules. Organs were harvested and immersion fixed in paraformaldehyde, paraffin embedded, sliced and stained with HE. Images are representative of 8 experiments.

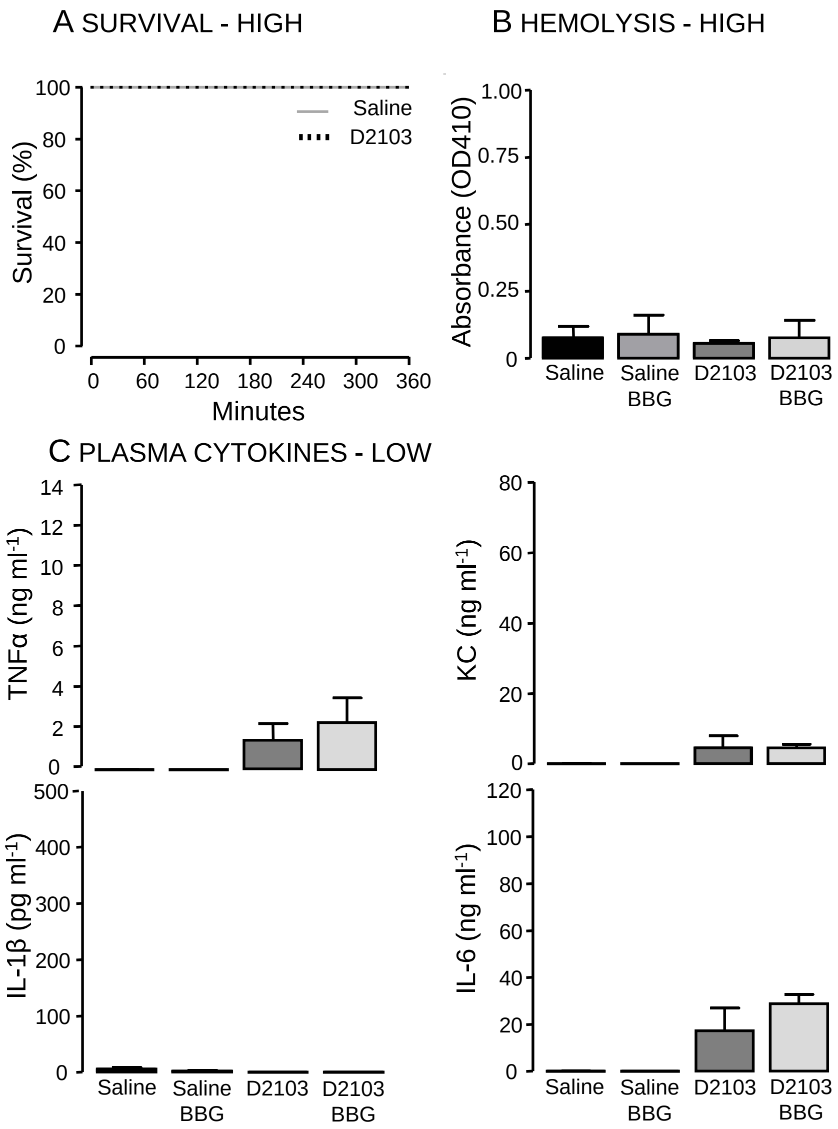

Figure S3. The non-haemolytic E. coli control strain: mortality, hemolysis and cytokine release.
The *E. coli* strain D2103 was injected *iv* into anaesthetised balb/cj mice at a concentration of 165 million bacteria. Two hours prior to ARD6 injection the animals were administred with the $P2X_7$ antagonist BBG or vehicle (50 mg kg-1, subcutaneously). (A) Kaplan-Meier plot shows survival during the 6 hours observation, n=6 for controls and 6 for D2103 ± BBG (B) Hemolysis in plasma 2.5 hours after D2103 injection, n=8 for all four groups. (C) Levels of the cytokines TNFα, KC, IL-1β and IL-6 2.5 hours after D2103 injection, n=6 for control and BBG and 6-8 for D2103±BBG.

PLASMA CYTOKINES - HIGH

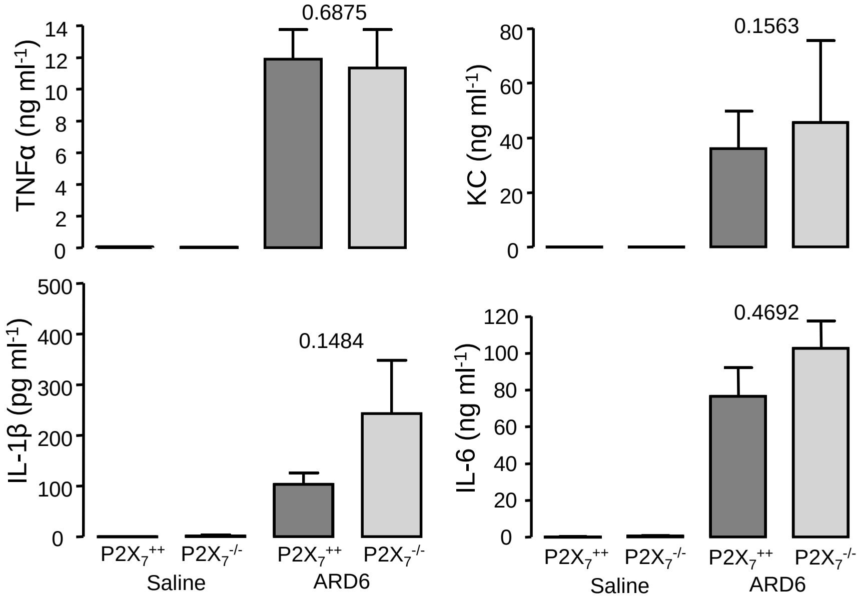

Figure S4. $P2X_7$ deficiency and *E. coli*-induced sepsis. ARD6 was injected into anaesthetized $P2X_7^{+/+}$ and $P2X_7^{-/-}$ mice (high dose - 165 million). Plasma TNFα, KC, IL-1β and IL-6 measured 2.5 hours after ARD6 injection, n=7-8 for controls and 10-12 for each genotypes.
